# Process optimization and nutritional profiling of gluten-free quinoa–rice couscous: amino acid and polyphenol characterization

**DOI:** 10.3389/fnut.2026.1861043

**Published:** 2026-07-10

**Authors:** Khadija El Hazzam, Kawtar Bettat, Mohamed Louay Metougui, Didier Bazile, Manal Mhada

**Affiliations:** 1 AgroBioSciences Department (AgBS), College of Agriculture and Environmental Science (CAES), Mohammed VI Polytechnic University, Ben Guerir, Morocco; 2 Faculty of Sciences and Techniques, Hassan II University, Mohammedia, Morocco; 3 Agricultural Innovation and Technology Transfer Center (AITTC), College of Agriculture and Environmental Science (CAES), Mohammed VI Polytechnic University, Ben Guerir, Morocco; 4 QUALINOA SAS de l'ESS, Claret, France; 5 CIRAD, UMR SENS, Montpellier, France; 6 UMR SENS, CIRAD, IRD, University Paul Valery Montpellier 3, University Montpellier, Montpellier, France

**Keywords:** quinoa couscous, gluten-free, processing parameters, D-optimal design, nutritional quality

## Abstract

**Introduction:**

Quinoa (Chenopodium quinoa Willd.) is internationally recognized for its exceptional nutritional profile, yet its incorporation into traditional cereal-based foods remains limited.

**Methods:**

This study developed and optimized a gluten-free couscous from quinoa and rice using D-optimal response surface methodology, evaluating the effects of quinoa fortification level (0-100%), steaming time (10-30min), and water volume (350-450 ml/kg) on techno-functional properties, proximate composition, mineral profile, bioactive compounds, and color.

**Results:**

Quinoa fortification percentage was the dominant factor across the response set, while steaming time and water volume acted as secondary modulators. The nutritional optimum converged on 100% quinoa, with processing parameters of 20min steaming and 400 ml/kg water. Compared with commercial wheat couscous and rice-only couscous, quinoa couscous showed enhanced water-holding and oil-holding capacity, up to five-fold higher potassium, three-fold higher iron, and three-fold higher total phenolic content. LC-MS/MS amino acid putative profiling detected 18-19 of 20 amino acids in quinoa couscous versus 14 in wheat and 11 in rice couscous, with enrichment in branched-chain amino acids and detection of lysine, the limiting amino acid of cereal proteins, which was undetected in rice couscous and present only at trace levels in wheat couscous. Quinoa couscous exhibited around five-fold greater polyphenol diversity than wheat couscous, including flavan-3-ols, chalcones, and hydroxycinnamic acids exclusive to quinoa formulations. Higher quinoa levels darkened the product, with color stabilizing at fortification levels above 75%.

**Discussion:**

Unlike previous work on alternative-grain couscous, this study combines process optimization with LC-MS profiling of amino acid and polyphenol composition, providing an integrated framework for gluten-free quinoa-rice couscous development. The results demonstrate that quinoa-based couscous provides a nutritionally superior, gluten-free alternative to conventional wheat couscous, with processing conditions that preserve the raw material's nutritional attributes.

## 1 Introduction

Food security, fundamental constituent of sustainable development, involves not just food availability but also afford safe and nutritious food (1). Globally, cereal-based foods are the main energy source; however, contemporary food systems face issues, i.e., chronic diseases, nutritional gaps, and intolerances (2). Consequently, there is increasing demand for diverse, nutrient-dense foods. In parallel, the prevalence of gluten-related disorders is rising, with celiac disease affecting approximately 1.4% of the population at a global and up to 3% in Mediterranean regions (2, 3). The demand for gluten-free products is growing due to the fact that a stringent gluten-free diet is presently the only effective treatment, which creates both technological and nutritional hurdles for food processing and product development (4).

Pseudocereals such as quinoa (*C. quinoa*) become a viable substitute for conventional cereals that contain gluten. Quinoa's remarkable nutritional profile, which includes a balanced amino acid composition, a high dietary fiber content, important lipids, and notable amounts of minerals and bioactive substances, has earned it recognition on a global scale (5, 6). In addition to its nutritional value, quinoa demonstrates exceptional agronomic adaptability, tolerating drought, salinity, and temperature variability, which increases its significance in relation to food security and climate change (7, 8). In Morocco, quinoa cultivation has expanded recently, offering opportunities to strengthen local food systems and rural economies. Nevertheless, despite increasing production, quinoa is predominantly marketed as a raw commodity, with limited value-added processing and product diversification (9).

One of the major constraints limiting the broader use of quinoa in food products is the presence of saponins, which impart bitterness and may affect digestibility and nutritional quality if not adequately removed (10, 11). Furthermore, transforming quinoa into traditional cereal-based foods involves technological adjustments to preserve its nutritional qualities while maintaining an acceptable texture, appearance, and cooking behavior. These adjustments emphasize how crucial it is to optimize processing conditions while creating novel quinoa-based products.

Couscous is a staple food in North Africa and a central component of the Moroccan diet, traditionally produced using wheat semolina. Its cultural significance and widespread consumption make couscous an attractive vehicle for nutritional enhancement. However, the dependence on wheat restricts nutritional diversity and excludes people with gluten intolerance (12). And creating a quinoa-based gluten-free couscous is an innovative approach to get around these restrictions. Previous studies have explored the incorporation of alternative grains into couscous. Nevertheless, limited research has focused on methodically improving processing parameters, such as water addition, steaming time, and ingredient ratios, that govern product quality, nutrient retention, and techno-functional properties (13, 14).

In food science, response surface methodology (RSM) is a potent statistical approach that is frequently used to predict and improve intricate processes involving numerous interacting factors. RSM can be used to determine ideal processing settings that minimize experimental cost and variability while balancing functional performance, technical viability, and nutritional quality (15).

The present work brings together three elements that have not, to our knowledge, been combined for gluten-free couscous: (i) D-optimal response surface optimization of the processing parameters (quinoa fortification level, steaming time, and water volume) that govern couscous quality, (ii) comprehensive techno-functional and proximate characterization across the resulting formulations, and (iii) untargeted LC-HRMS profiling of amino acid diversity and polyphenol composition, complemented by mineral profiling. Earlier work on quinoa- or alternative-grain-enriched couscous has mainly addressed composition or cooking quality (13, 14), and quinoa polyphenols have been profiled by untargeted MS in raw grain or sprouts (16, 17), but the integration of process optimization with compound-level nutritional profiling for a gluten-free couscous matrix is, to our knowledge, addressed here for the first time.

Therefore, the objective of this study is the development and optimization of a gluten-free couscous formulation using quinoa and rice, applying RSM. The effects of processing parameters on techno-functional properties, proximate composition, mineral profile, bioactive compounds, and color characteristics were systematically investigated. This work aims to contribute to the development of nutritionally enhanced, culturally relevant gluten-free foods and to provide a scientifically robust framework for optimizing quinoa-based couscous production.

## 2 Materials and methods

### 2.1 Raw materials

Quinoa (*C. quinoa*, Puno variety) grains were obtained directly from a local producer in Berrechid, Morocco. The grains were harvested in 2024. The Puno variety was selected because it is among the quinoa varieties most widely adopted by Moroccan producers, is well adapted to Mediterranean and semi-arid growing conditions, and has been characterized in previous studies, allowing comparison of its nutritional and processing behavior with published data (18).

Commercial white rice grains (brand: Presto, round rice) were purchased from a local market. The rice was produced in Morocco. All raw materials were stored under dry conditions at 20–25 °C before processing.

Commercial medium wheat couscous from Alitkane brand produced in Casablanca, Morocco, was used as a standard sample for comparison.

### 2.2 Preparation of couscous ingredients

Quinoa and rice grains were processed separately to obtain semolina and flour, which were subsequently used to formulate couscous.

Before milling, quinoa grains were polished to remove the outer saponin-containing layer using a custom-built mechanical grain polisher designed by the Moroccan quinoa producer. After polishing, quinoa grains were subjected to a milling process to obtain quinoa semolina using a wheat roller mill with grooved steel rollers, adjusted to produce coarse particles. The resulting material was sieved through a 1.0 mm mesh sieve to separate the semolina fraction. Further on, a spice mill (DAMAY, 250 g capacity) was used for the preparation of rice semolina and flour with sizes of 1 mm and < 250 μm, respectively. The quinoa flour was obtained as rice flour prepared using quinoa semolina.

Quinoa and rice fractions were stored in airtight containers at 20–25 °C until couscous processing.

### 2.3 Couscous processing

**Figure 1:**
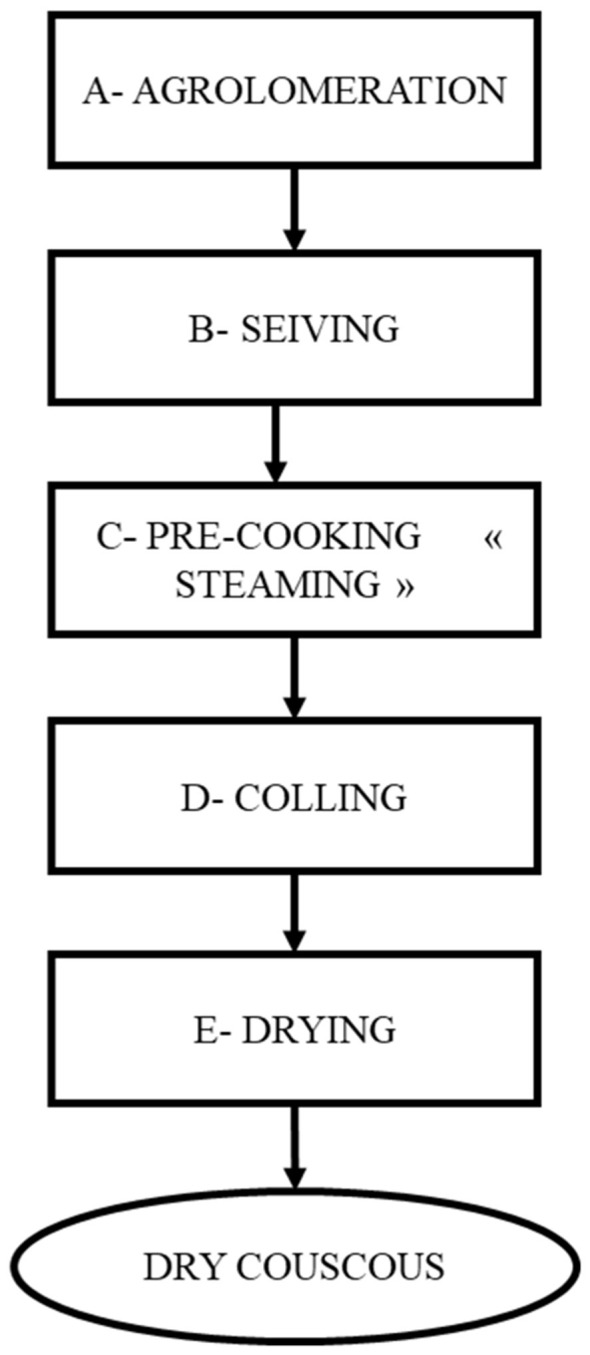
Moroccan traditional couscous processing diagram.

Couscous was produced following a traditional Moroccan processing method, representative of household and local cooperative practices. The processing steps and operational conditions were defined based on established traditional couscous-making procedures commonly used and subsequently standardized to allow for controlled variation of processing parameters. The process consisted of six steps (Figure 1):

#### 2.3.1 Agglomeration

Agglomeration was performed by manually mixing semolina with gradual addition of distilled water and the corresponding flour fraction, as per the experimental design. The ingredients were mixed and rolled until homogeneous granules were formed. The amount of water added during agglomeration was adjusted according to the experimental design (Figure 1A).

#### 2.3.2 Sieving

Following agglomeration, the couscous granules were sieved using a 2 mm sieve to obtain uniformly sized particles (Figure 1B).

#### 2.3.3 Steaming

The sieved couscous granules were precooked by steaming using a traditional couscous steamer placed over boiling water. Steaming was carried out at atmospheric pressure for 10, 20, or 30 min, depending on the experimental conditions, using a constant heat source (Figure 1C).

#### 2.3.4 Cooling

After steaming, the couscous was spread and allowed to cool at ambient temperature (25 ± 3 °C) to stabilize granule structure and prevent clumping before drying (Figure 1D).

#### 2.3.5 Drying

The precooked couscous was naturally air-dried on a cotton fabric for 48 h at ambient temperature until a stable dry product was obtained (Figure 1E).

After drying, couscous samples intended for physicochemical and nutritional analyses were ground into a fine powder (< 250 μm) using a laboratory grinder (DAMAI^®^ GRINDER, HC-250, China) and stored at 4 °C until analysis.

### 2.4 Optimization of the couscous processing using response surface methodology

The couscous processing conditions were optimized using response surface methodology (RSM) to evaluate the combined effects of formulation and processing parameters on product quality. A D-optimal experimental design was applied to model the relationships between selected independent variables and multiple response variables. Three independent factors were selected based on preliminary trials and traditional couscous processing practices: Percentage of quinoa fortification (A, %), steaming time (B, min), and volume of water used during the agglomeration step (C, ml per kg of ingredients). Each factor was investigated at three levels, as presented in (Table 1).

**Table 1:** Levels of selected factors.

Factors		Level−1	Level 0	Level 1
A:	Quinoa fortification percentage (%)	0	50	100
B:	**Steaming time (min)**	10	20	30
C:	**Volume of water for 1 kg (ml)**	350	400	450

The design generated 17 experimental runs to adequately describe the experimental domain while minimizing the number of trials (Supplementary Table SI). The response variables evaluated included techno-functional properties (water holding capacity, oil holding capacity, and swelling capacity), proximate composition (protein, carbohydrates, fat, ash, and moisture), mineral content (P, K, Ca, Mg, Fe, and Zn), total phenolic content, and soluble sugars.

### 2.5 Techno-functional properties and physico-chemical analysis

#### 2.5.1 Techno-functional properties

##### Water holding capacity (WHC)

The water-holding capacity (WHC) of the couscous formulas was measured according to the method of Pellegrini et al. (19). WHC was expressed as the g of water held per g of flour.

##### Oil holding capacity (OHC)

Oil holding capacity (OHC) was measured following the method of Pellegrini et al. (19), with modifications followed by El Hazzam et al. (20). OHC was determined using the following equation:

*OHC (g/g)* = *(P*_0_*-P*_1_*)/m*_0_

Where *m0* denotes 1 g of the sample, *P0* represents the weight of 10 ml of corn oil, and *P1* corresponds to the weight of the unabsorbed corn oil.

##### Swelling capacity (SC)

A mixture of 1 g of the sample with 10 ml of distilled water was heated at 95 °C for 30 min. After cooling to room temperature, it was centrifuged at 6,000 rpm for 15 min. Finally, the supernatant was removed, and the volume of the residue was recorded. SC was expressed as the volume increase in ml/g of couscous (21).

#### 2.5.2 Proximate composition

The raw material and couscous samples underwent comprehensive analysis to determine their moisture (method 44–19), ash (method 08–01), and fat content. The analysis followed the AOAC standard methods (22). For the protein content, the Kjeldahl method using a Kjeltec 2,300 autoanalyzer was done in accordance with the official AOAC International analytical procedures (23). The total nitrogen content was measured, and protein content was calculated using the specific nitrogen-to-protein conversion factors for quinoa and rice, 5.75 and 5.95, respectively (24). All measurements were performed in triplicates.

#### 2.5.3 Total phenolic, saponin, and soluble sugar content

##### Preparation of the extracts

The extracts were prepared by mixing 1 g of each sample with 10 ml of an ethanol–water solution (50:50, v/v). The mixtures were shaken on an orbital shaker for 72 h at 25 ± 2 °C, then centrifuged at 4,000 rpm for 15 min at 4 °C, filtered, and adjusted to a final volume of 10 ml using the extraction solution.

##### Total phenolic content (TPC)

Total phenolic content was determined using the Folin–Ciocalteu method, as described by Mhada et al. (25) and adapted to microplate format. 20 μl of the extract was mixed with 100 μl of Folin–Ciocalteu reagent (HC97724201, Merck), incubated for 5 min, and 80 μl of 7.5% anhydrous sodium carbonate solution (Na_2_CO_3_) was added. After 30 min of incubation, the absorbance was measured at 750 nm using a microplate reader (BMG Labtech Fluostar Omega). Gallic acid (GA) was used as a reference standard (0–100 μg/ml; R^2^ = 0.9972), and the results were expressed as mg of GA equivalents per g of sample (mg GA/g).

##### Saponins content

Saponin content was quantified using the method of Irigoyen & Giner (26). The absorbance was measured at 528 nm using a microplate reader (BMG Labtech Fluostar Omega); the oleanolic acid (OA) was used as a reference standard (0–100 μg/ml; R^2^ = 0.9986), and the results were expressed as mg of OA equivalent per g of sample (mg OA/g).

##### Soluble sugar content

Soluble sugars were determined using the anthrone method. A volume of 1 ml extract was added to 2 ml of anthrone reagent (1 g/L anthrone in 96% sulfuric acid). After vortexing, the mixture was placed in a water bath at 95 °C for 10 min, cooled on ice, and the absorbance was measured at 630 nm using a microplate reader. Glucose was used as the calibration standard (R^2^ = 0.9921), and results were expressed as mg glucose equivalents per gram of dry matter (mg G/g).

#### 2.5.4 Mineral profile

The mineral composition of the samples was determined following the method described by Mhada et al. (25). Briefly, dried couscous samples were ground to a fine powder. Approximately 500 ± 0.5 mg of each sample was weighed in triplicate and transferred into digestion vessels. 7.5 ml of concentrated nitric acid (65% HNO3) was added to each vessel, and the samples were left uncapped for 20 min to allow initial reaction and pressure release. After this step, they were sealed, placed in a microwave digestion rotor, and digested at 90 °C for 2 h. Once digestion was complete, the volume was adjusted to 50 ml using deionized water. An aliquot of each digested sample was further diluted to 10 ml before analysis. The concentrations of phosphorus (*P*), potassium (K), calcium (Ca), magnesium (Mg), iron (Fe), and zinc (Zn) were determined using inductively coupled plasma optical emission spectroscopy (ICP-OES, Agilent 5,110, United States). Quantification was performed using single-element ICP standards (TraceCERT, 1,000 mg/L in nitric acid, Merck). Calibration standards were prepared from stock solutions and stored at 4 °C. Mineral contents were expressed as percentages for macroelements (P, K, Ca, Mg) and mg/kg dry matter for trace elements (Fe, Zn).

#### 2.5.5 Amino acid profile

The isolation of protein and derivatization of amino acids were performed according to the methods of (27–29). Briefly, 10 g of defatted sample was added to 100 g of distilled water, and the pH was adjusted to 10 with 2 M NaOH. The mixture was stirred at 35 °C for 90 min. After stirring, the mixture was centrifuged, and the supernatant was collected. Then, protein precipitation was performed by adding 0.1 M HCl until the pH reached 4.4. A second centrifugation was performed at 4,000 rcf for 20 min, and the pellet was collected. A 6 M HCl solution was added to the pellet, and the glass tubes were placed in an oven at 110 °C for 24 h. Then, a 6 M NaOH solution was added to each tube to adjust the pH to 4.5. The extracts were stored at−20 until analysis.

#### 2.5.6 LC–HRMS analysis

Analyses were performed using a Thermo Scientific™ Vanquish™ Flex UHPLC system coupled to an Orbitrap Exploris™ 240 high-resolution mass spectrometer (Thermo Fisher Scientific, Waltham, MA, USA).

A Hypersil HPLC column (1.9 μm, 2.1 mm × 50 mm) was used for chromatographic separation. The mobile phase consisted of 0.1% formic acid in water (LC-MS grade) for the phase A and 0.1% formic acid in acetonitrile (LC-MS grade) for the phase B. Elution was performed using the following linear gradient: 0% B at 0 min, 99% B at 22 min, kept at 99% B until 27 min, returned to 0% B at 27.1 min, and re-equilibrated at 0% B until 30 min. The flow rate was set at 0.5 ml/min, and the sample compartment temperature was maintained at 25 °C.

Mass spectrometric detection was performed in electrospray ionization mode (H-ESI II) with spray voltages of 3.7 kV and 2.5 kV in positive and negative ionization modes, respectively. The ion transfer tube temperature was set to 325 °C and the vaporizer temperature to 350 °C. Data were acquired in Full MS/ddMS^2^ mode at a resolution of 60,000 (at m/z 200).

Data acquisition was performed using Thermo Scientific™ TraceFinder™ software, and compound detection and data processing were carried out with Thermo Scientific™ Compound Discoverer™ 3.3.

Compound identification was performed by matching experimental spectra against the mzCloud spectral library. The identification corresponds to level 2 (putative annotation by library match) according to the Metabolomics Standards Initiative reporting criteria (30). Relative compound abundance was scored on a semi-quantitative scale from “–” (not detected) to “+++++” (very high), based on peak area ratios normalized to the maximum signal observed for each compound across all extracts (Table 2).

**Table 2:** Semi-quantitative scoring legend.

Symbol	Meaning	Relative concentration
–	Not detected	0%
**+**	Trace	< 10% of max
**++**	Low	10–25% of max
**+++**	Moderate	25–50% of max
**++++**	High	50–75% of max
**+++++**	Very high	> 75% of max

#### 2.5.7 Color measurements

Color characteristics of couscous samples were measured using a Minolta CM-5 spectrophotometer (Konica Minolta, Osaka, Japan) operating in the CIELab color space. Measurements were conducted using illuminant D65, SCI mode, and a 10° standard observer. A low-reflectance glass plate (Minolta CR-A51/1829-752) was placed between the sample and the measurement port to ensure consistent surface conditions. The color parameters recorded were lightness (L^*^), redness-greenness (a^*^), and yellowness-blueness (b^*^) (31).

#### 2.5.8 Visual appearance

The visual appearance of couscous granules was qualitatively assessed using digital images of samples formulated with varying levels of quinoa fortification. Images were captured at room temperature using a Huawei Y8p smartphone camera (Huawei, China) under laboratory light.

Photographs were used solely for visual comparison of color and granule appearance and were not subjected to quantitative image analysis.

### 2.6 Statistical analysis

All experimental results were expressed as mean ± standard deviation (SD). For experiments involving fixed processing conditions and comparison of quinoa fortification levels, statistical differences between means were evaluated using analysis of variance (ANOVA) followed by Tukey's honestly significant difference (HSD) *post hoc* test at a 95% confidence level (*p* < 0.05). These statistical analyses were performed using RStudio software (version 2024.12.0+467).

For the optimization study, data obtained from the response surface methodology were analyzed by fitting linear or quadratic polynomial models, depending on the significance of model terms. Model adequacy was assessed using analysis of variance (ANOVA), coefficients of determination (R^2^), adjusted R^2^, predicted R^2^, lack-of-fit tests, and residual analysis. Numerical optimization of processing parameters was conducted using a desirability function approach to identify optimal conditions. All response surface modeling, statistical analyses related to RSM, and optimization procedures were performed using Design-Expert software, version 13 (Stat-Ease Inc., Minneapolis, MN, USA).

## 3 Results

### 3.1 Physico-chemical characterization of couscous ingredients

The nutritional quality of quinoa semolina (QS) and rice flour (RF), used as raw ingredients for couscous formulation, was evaluated based on proximate composition, mineral profile, saponin content, total phenolic content, color parameters, and techno-functional properties (Oil Holding Capacity (OHC), Water Holding Capacity (WHC), and Swelling Capacity (SC)). The results are presented in Table 3.

**Table 3:** Physico-chemical characterization of quinoa and rice used to formulate couscous.

Physico-chemical analysis	Quinoa semolina	Rice flour
Proximate composition		
Moisture (%)	13.68 ± 0.35a	13.72 ± 0.30a
Protein (%)	7.91 ± 0.29a	7.78 ± 0.28a
Fat (%)	1.81 ± 0.00a	0.94 ± 0.00b
Ash (%)	1.30 ± 0.18a	0.28 ± 0.03b
Carbohydrates (estimate)	75 ± 0.24a	73.84 ± 0.36b
Soluble sugar (mg G/g)	8.69 ± 0.17a	4.46 ± 0.53b
Mineral profile		
P (%)	0.14 ± 0.01a	0.11 ± 0.00b
K (%)	0.46 ± 0.01a	0.09 ± 0.01b
Ca (%)	0.04 ± 0.00a	0.02 ± 0.00b
Mg (%)	0.07 ± 0.00a	0.03 ± 0.00b
Fe (mg/kg)	38.37 ± 9.35a	7.91 ± 0.99b
Zn (mg/kg)	13.56 ± 0.27a	13.93 ± 0.14a
Bioactive compounds		
Saponin content (mg OA/g)	0.53 ± 0.08a	0.17 ± 0.04b
TPC (mg GAE/g)	0.45 ± 0.09a	0.16 ± 0.01b
Color parameters		
L^*^ (Lightness)	82.15 ± 0.08b	90.86 ± 0.08a
a^*^ (redness - greenness)	1.29 ± 0.06a	−0.22 ± 0.03b
b^*^ (yellowness - blueness)	15.22 ± 0.16a	7.99 ± 0.22b
Techno-functional properties		
Water holding capacity (WHC)	1.21 ± 0.04a	0.91 ± 0.04b
Oil holding capacity (OHC)	1.33 ± 0.03a	0.75 ± 0.05b
Swelling capacity (SC)	4.07 ± 0.06a	3.93 ± 0.06b

The asterisks for L^*^, a^*^, and b^*^ are standard, non-optional components of the International Commission on Illumination (CIE) color space notation.

QS exhibited higher values than RF for most nutritional parameters. Regarding proximate composition, QS contains approximately twice the fat content (1.81% vs. 0.94%) and more than four times the ash content (1.30% for QS vs. 0.28% for RF). Notably, QS contained approximately twice the soluble sugar of rice (8.69 mg G/g vs. 4.46 mg G/g). In contrast, no significant differences were observed in moisture or protein content.

For the mineral profile, quinoa showed substantially higher contents of the most analyzed elements. Potassium content was approximately five times higher in QS (0.46%) than in RF (0.09%). Similarly, quinoa contained twice the calcium (0.04% vs. 0.02%), more than twice the magnesium (0.07% vs. 0.03%), and nearly five times the iron content (38.37 mg/kg vs. 7.91 mg/kg). While zinc content was similar (13.56 mg/kg in quinoa vs. 13.93 mg/kg in rice).

QS also contained higher levels of bioactive compounds. TPC was approximately three times higher in QS (0.45 mg GAE/g) compared to rice (0.16 mg GAE/g). Saponin content was also higher in quinoa (0.53 mg OA/g) than in rice (0.17 mg OA/g), although the amount of saponins was below the consumption threshold (0.11%) in bought cases.

Regarding color parameters, rice grains were significantly lighter than quinoa, with L^*^ values of 90.86 and 82.15, respectively. The a^*^ value was positive for quinoa (1.29), indicating a slight red tone, whereas rice exhibited a slightly negative value (−0.22), indicating a neutral to faint greenish hue. For the b^*^ parameter (yellowness–blueness), quinoa showed a substantially higher value (15.22) compared to rice (7.99), indicating a more intense yellow coloration.

Regarding techno-functional properties, QS exhibited higher values for all measured parameters. Water-holding capacity was 1.21 g/g for quinoa, compared to 0.91 g/g for rice. Oil-holding capacity was 1.33 g/g in quinoa, compared with 0.75 g/g in rice. Swelling capacity was slightly higher in quinoa (4.07 ml/g) than in rice (3.93 ml/g).

### 3.2 Couscous processing optimization using response surface methodology

#### 3.2.1 Model assessment and data analysis

The optimization of couscous processing was carried out using a D-optimal design within the response surface methodology (RSM) framework. Three independent factors were investigated: quinoa fortification percentage (A, %), steaming time (B, min), and water volume (C, ml/kg). The response variables included techno-functional properties (oil holding capacity (OHC), water holding capacity (WHC), and swelling capacity (SC)), proximate composition (moisture, protein, fat, ash, and carbohydrates), total phenolic content (TPC, mg GAE/g dry matter), soluble sugar content (mg G/g dry matter), and mineral content (K, Ca, Mg, and Fe). A total of 17 experimental runs were generated by the design (Supplementary Table S1). The use of a D-optimal design enabled efficient exploration of the experimental domain while accounting for practical constraints associated with traditional couscous processing.

The analysis of variance (ANOVA) results provides an overview of model performance and statistical significance for the different responses, evaluated using linear or quadratic regression models. As summarized in Table 4, quadratic models were appropriate for OHC, WHC, SC, moisture and TPC, with *F*-values of 34.08, 28.35, 16.55, 11.02, and 46.21, respectively. And linear models adequately described fat, ash, carbohydrates, soluble sugar, and minerals (P, K, Ca, Mg, Fe, and Zn), with the highest *F*-values of 1,450.58 for potassium. All models are statistically significant except for the response protein, for which no significant model terms were obtained, indicating that the model has no predictive power because none of the tested factors have a statistically significant effect on the protein content.

**Table 4:** ANOVA Analysis of response surfaces.

Responses		Techno-functional properties				Proximate composition (%)				TPC	Soluble Sugar (mg/g)	Mineral profile					
		OHC	WHC	SC	Moisture	Protein	Fat	Ash	Carbohyd-rates			*P* (%)	K (%)	Ca (%)	Mg (%)	Fe (mg/kg)	Zn (mg/kg)
	Process order	Q	Q	Q	Q	–	L	L	L	Q	L	L	L	L	L	L	L
Model	***F*** **–value**	34.080	28.350	16.550	11.02	–	70.780	25.460	9.140	46.210	27.130	12.800	1,450.580	331.500	27.130	21.400	5.250
	***P*** **–value**	< 0.0001^***^	0.0001^***^	0.0006^***^	0.0023^***^	–	< 0.0001^***^	< 0.0001^***^	0.0016	< 0.0001^***^	< 0.0001^***^	0.0004	< 0.0001^***^	< 0.0001^***^	< 0.0001^***^	< 0.0001^***^	0.0136
A	***F*** **–value**	33.660	82.900	1.350	22.180	–	210.880	73.210	24.620	324.950	80.750	35.920	4,351.620	994.500	80.750	63.640	1.110
	***P*** **–value**	0.0007^***^	< 0.0001^***^	0.2828	0.0022^**^	–	< 0.0001^***^	< 0.0001^***^	0.0003^***^	< 0.0001^***^	< 0.0001^***^	< 0.0001^***^	< 0.0001^***^	< 0.0001^***^	< 0.0001^***^	< 0.0001^***^	0.3120
B	***F*** **–value**	0.556	35.060	0.0118	0.2274	–	0.0156	2.460	0.6086	6.280	0.425	0.2494	0.094	6.280	0.425	0.030	2.090
	***P*** **–value**	0.480	0.0006	0.9167	0.6480	–	0.9025	0.1408	0.4493	0.041^*^	0.526	0.6258	0.764	1	0.527	0.866	0.1719
C	***F*** **–value**	0.575	44.560	0.0048	20.070	–	1.440	0.7201	2.180	15.350	0.206	2.240	0.023	15.350	0.206	0.535	12.55
	***P*** **–value**	0.473	0.0003^***^	0.9469	0.0029^**^	–	0.2515	0.4115	0.1634	0.007^**^	0.657	0.1579	0.881	1	1	0.478	0.0036
AB	***F*** **–value**	23.470	40.170	25.900	7.650	–	–	–	–	6.360	–	–	–	–	–	–	–
	***P*** **–value**	0.002^***^	0.0004^***^	0.0014^**^	0.0278^*^	–	–	–	–	0.040^*^	–	–	–	–	–	–	–
AC	***F*** **–value**	7.190	27.780	7.700	2.300	–	–	–	–	47.180	–	–	–	–	–	–	–
	***P*** **–value**	0.032^*^	0.0012^**^	0.0275^*^	0.1734	–	–	–	–	0.0002^***^	–	–	–	–	–	–	–
BC	***F*** **–value**	0.032	9.220	1.570	14.940	–	–	–	–	1.120	–	–	–	–	–	–	–
	***P*** **–value**	0.863	0.0190^*^	0.2498	0.0062^**^	–	–	–	–	0.325	–	–	–	–	–	–	–
A^2^	***F*** **–value**	138.240	6.960	105.180	1.280	–	–	–	–	10.690	–	–	–	–	–	–	–
	***P*** **–value**	< 0.0001^***^	0.0335^*^	< 0.0001^***^	0.2958	–	–	–	–	0.0137^*^	–	–	–	–	–	–	–
B^2^	***F*** **–value**	6.700	0.2329	0.9461	14.810	–	–	–	–	1.220	–	–	–	–	–	–	–
	***P*** **–value**	0.036^*^	0.6441	0.3631	0.0063^**^	–	–	–	–	0.307	–	–	–	–	–	–	–
C^2^	***F*** **–value**	77.520	7.150	8.020	17.310	–	–	–	–	2.630	–	–	–	–	–	–	–
	***P*** **–value**	< 0.0001^***^	0.0319^*^	0.0253^*^	0.0042^**^	–	–	–	–	0.149	–	–	–	–	–	–	–
Lack of fit	***F*** **–value**	0.779	0.317	0.603	0.152	0.316	1.630	0.652	0.665	5.120	0.813	0.590	1.639	0.078	0.561	2.584	0.983
	***P*** **–value**	0.564	0.814	0.647	0.923	0.946	0.335	0.727	0.719	0.074	0.636	0.769	0.334	0.999	0.784	0.187	0.552

A: % of fortification; %, B: Steaming time; min, C: volume of water; mL/kg of dry matter, OHC: oil holding capacity (g/g), WHC: water holding capacity (g/g), SC: swelling capacity (mL/g), TPC: total phenolic content (mg GA/g), Q: Quadratic model, L: Linear model, ^*^**: Significant at P < 0.001, ^**^: Significant at P < 0.01, ^*^: Significant at P < 0.05.

Quinoa fortification percentage (factor A) was consistently significant across all responses (*P* < 0.001), except for SC and Zn with low *F*-values (1.35 and 1.11) and *p*-values of 0.28 and 0.31, respectively. Whereas steaming time (factor B) showed limited effects, with a significant *p*-value only for WHC and TPC; similarly, water volume (factor C) indicated a comparatively weaker influence, with significant effects on WHC, moisture, TPC, and Zn. For the quadratic models, significant interaction effects were observed. The interaction between quinoa fortification percentage and steaming time (AB) has a significant effect on all quadratic models; the interaction AC also has a significant effect on all responses except moisture. The interaction between BC had a lesser effect, with significant impacts only on WHC and moisture. Regarding squared terms, A^2^ and C^2^ showed significant values for the majority of quadratic models.

The responses described by the linear models were primarily influenced by quinoa fortification level, reflecting substantial compositional differences between quinoa and rice raw materials, except for Zn, which was more affected by the volume of water.

The lack-of-fit tests for the validated models were non-significant (*P* > 0.05), confirming that the fitted models adequately represented the experimental data. Overall, these models demonstrated good predictive performance and were suitable for interpretation and optimization.

**Figure 2:**
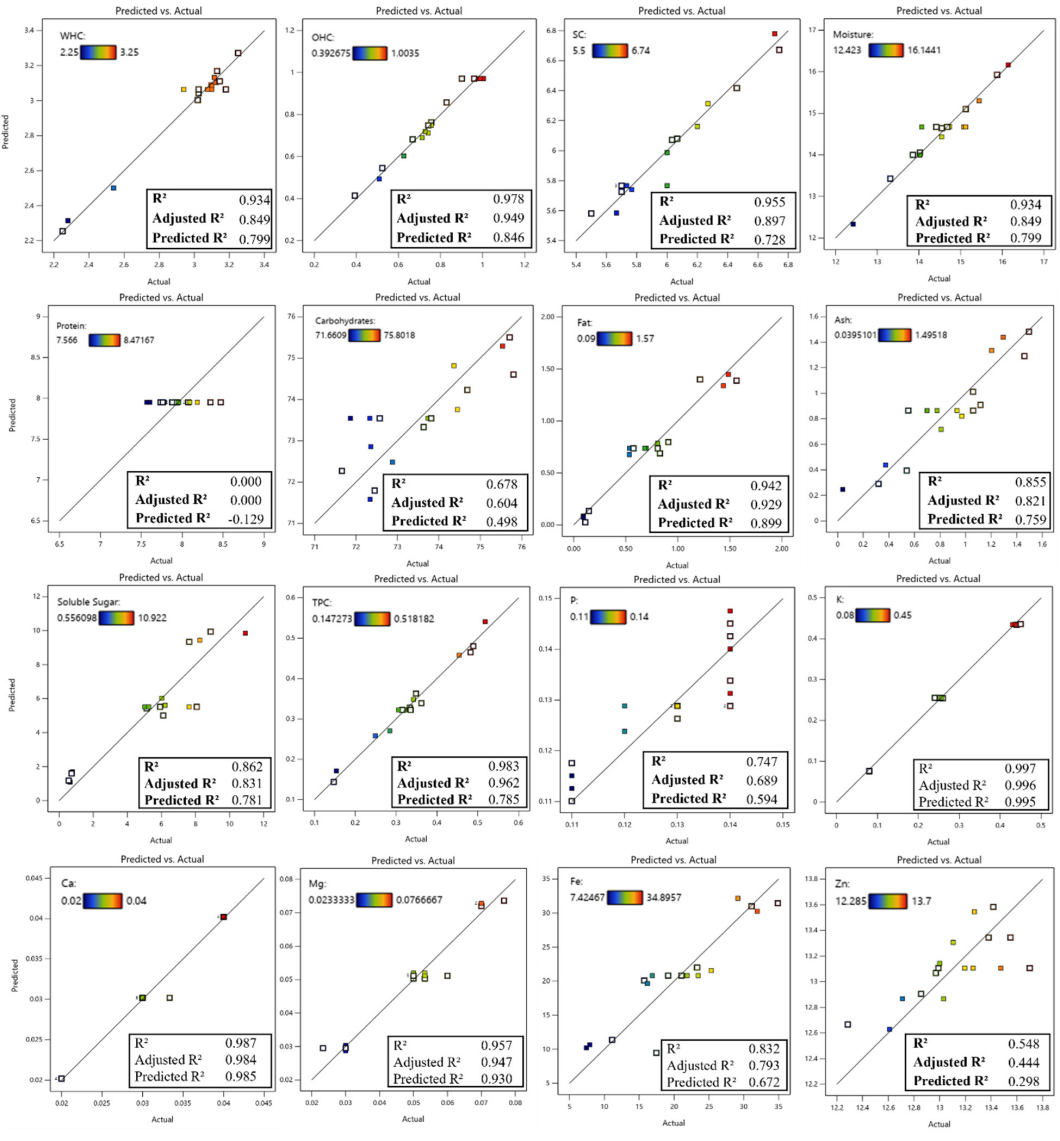
Actual and predicted values for the quadratic and linear models of techno-functional properties (OHC, WHC, and SC), TPC, soluble sugar, protein content, carbohydrates, moisture, fat, ash, and mineral contents (P, K, Ca, Mg, and Fe, and Zn).

Figure 2 presents the relationships between actual and predicted values for the responses. The close agreement between experimental and predicted values indicates good model accuracy, with differences between adjusted and predicted R^2^ values below 0.2 for all validated models. Except for protein content, a negative predicted R^2^ was noted, which literally means that the model predicts the data worse than if simply the average for this response was considered.

Residual analyses of the responses (Supplementary Figure S1) showed random, symmetrical distributions around zero, with no apparent trends or funnel-shaped patterns, confirming homoscedasticity and the absence of influential outliers, further supporting the adequacy of the regression models.

#### 3.2.2 Response surface analysis and process optimization

Response surface methodology was applied to visualize and interpret the combined effects of quinoa fortification percentage (A), steaming time (B), and water volume (C) on the response variables. The evaluation of this effect was done based on regression coefficients and predicted trends; the corresponding regression equations are presented in Table 5. The quinoa fortification percentage revealed a positive impact on all parameters studied. For the second factor, positive effects were observed for SC, soluble sugar, potassium, and magnesium. In contrast, a negative correlation was observed between the steaming time and the other responses. Similarly, the volume of water negatively impacted the majority of responses. For calcium and magnesium, no effect was observed on steaming time or water volume. For protein content, none of the studied factors had an impact, as confirmed by the fitting analysis.

**Table 5:** Final equation in terms of actual factors.

Responses	Equations
WHC	=3.06+0.2412 × A-0.1569 × B-0.1769 × C+0.2375 × AB+0.1975 × AC-0.1138 × BC-0.0964 × A^2^-0.0176 × B^2^-0.0976 × C^2^
OHC	=0.9712+0.0834 × A-0.0107 × B-0.0109 × C+0.0985 × AB+0.0545 × AC-0.0036 × BC-0.23309594625 × A^2^-0.0513 × B^2^-0.1746 × C^2^
SC	=5.77-0.0492 × A+0.0046 × B-0.0029 × C-0.3042 × AB+0.1658 × AC-0.0750 × BC+0.5975 × A^2^+0.0567 × B^2^-0.1650 × C^2^
Moisture	=14.67-0.5918 × A-0.0599 × B+0.5629 × C-0.4916 × AB+0.2693 × AC-0.6868 × BC-0.1957 × A^2^+0.6665 × B^2^-0.7206 × C^2^
Protein	=7.95
Carbohydrates	=73.54-1.51 × A-0.2375 × B-0.4497 × C
Fat	=0.7375+0.6576 × A-0.0057 × B-0.0543 × C
Ash	=0.8646+0.5223 × A-0.0957 × B-0.0518 × C
TPC	= 0.3222+0.1291 × A−0.0179 × B-0.0281 × C+0.0255 × AB+0.0696 × AC-0.0107 × BC+0.0323 × A^2^-0.0109 × B^2^+0.0160 × C^2^
Soluble sugar	= 5.5160+4.1245 × A+0.2993 × B +0.2084 × C
P	=0.1288+0.0150 × A-0.0012 × B-0.0038 × C
K	=0.2553+0.1796 × A+0.0008 × B+ 0.0004 × C
Ca	= 0.0302+ 0.01 × A+0 × B+0 × C
Mg	=0.0512+0.0217 × A + 0.0008 × B+0 × C
Fe	=20.8208+10.3991 × A-0.2238 × B-0.9534 × C
Zn	=13.11+0.1006 × A-0.1383 × B-0.3390 × C

A: % of fortification; %, B: Steaming time; min, C: volume of water; mL/kg of dry matter. OHC: oil holding capacity (g/g), WHC: water holding capacity (g/g), SC: swelling capacity (mL/g), TPC: total phenolic content (mg GA/g).

**Figure 3:**
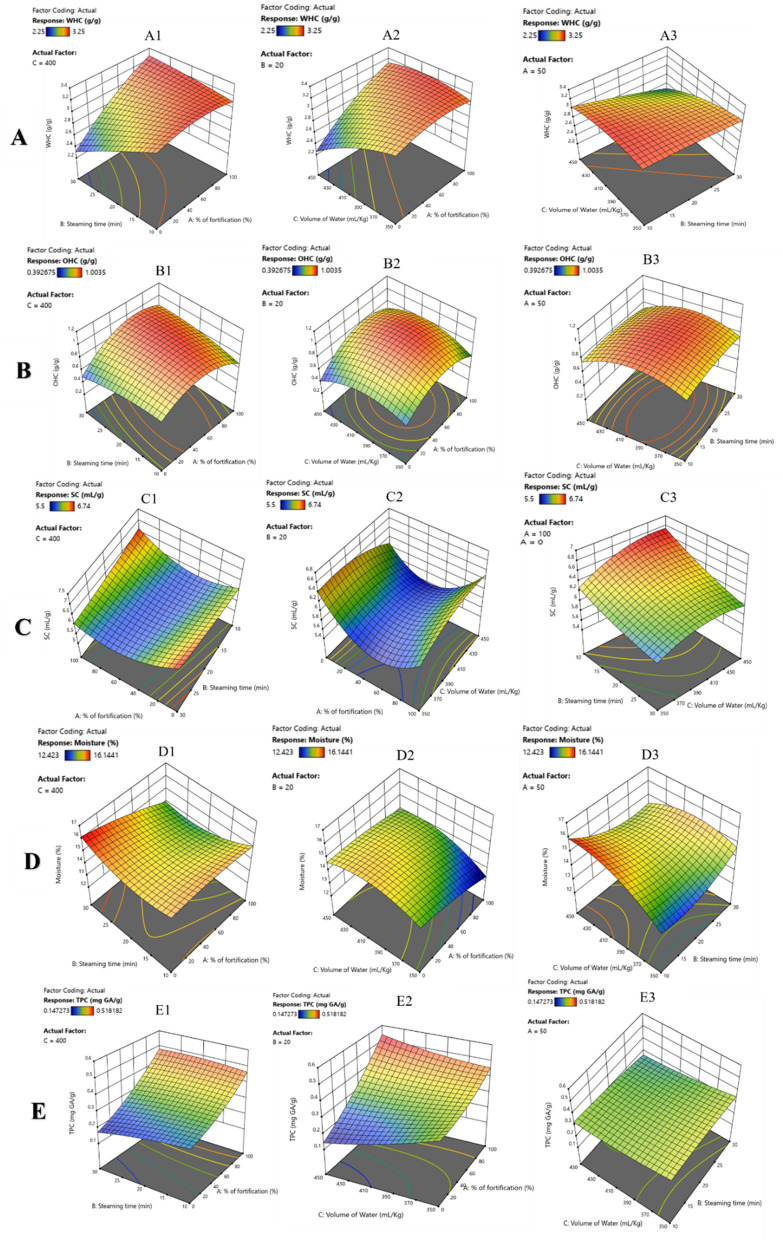
Contour response surface (3D) of the interaction effect between parameters: % offortification (%), Steaming time (min), and volume of water (ml/kg of dry matter) on responses with quadratic models [WHC **(A)**, OHC **(B)**, SC **(C)**, moisture **(D)**, and TPC **(E)**].

Three-dimensional response surface plots were generated for responses described by quadratic models (OHC, WHC, SC, moisture, and TPC) and illustrated in Figure 3, showing the effect of the interactions between two processing variables when the third factor is held constant at its central level (A = 50%, B = 20 min, C = 400 ml/kg) on these responses. Water Holding Capacity (WHC) increased progressively with higher fortification levels and longer steaming times, exhibiting a near-linear, additive behavior with no significant factor interaction. With maximum values reached under intermediate-to-high fortification and steaming settings (Figures 3A, A1, A2, A3). For OHC, quadratic effects and significant two-factor interactions were exhibited (A and C), suggesting an optimal formulation domain rather than a linear response with saddle-shaped surfaces; intermediate to high values of both fortification level and steaming time yielded higher OHC (Figures 3B, B1, B2, B3). These trends are consistent with the regression coefficients and interaction terms reported in Table 4.

Swelling Capacity (SC) exhibited a complex response, characterized by steep convex surfaces and a distinct optimal zone at intermediate water volume and fortification level, indicating that excessive fortification partially disturbs the starch swelling network (Figures 3C, C1, C2, C3). Moisture content followed predominantly linear trends, increasing with higher water volume and prolonged steaming, confirming enhanced water uptake during hydrothermal processing (Figures 3D, D1, D2, D3). Total phenolic content increased strongly with increasing quinoa fortification percentage, confirming quinoa's dominant contribution to phenolic compounds (Figures 3E, E1, E2, E3). These results demonstrate that the formulation ingredients are the primary drivers of phenolic content and that processing conditions can modulate phenolic retention slightly, which indicates the good thermal stability of the bioactive phenolic compounds. The contour plots for OHC and SC confirmed significant quadratic effects, validating the use of a second-order RSM model.

**Figure 4:**
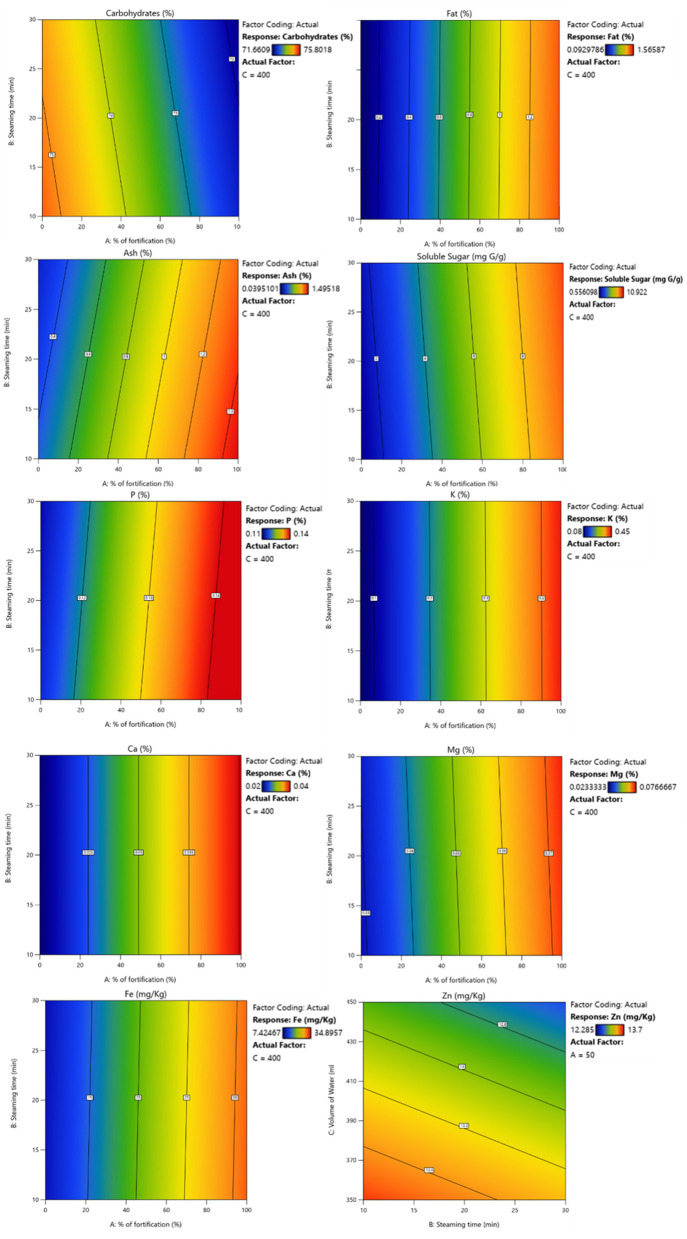
Contour response surface (2D) of the interaction effect between parameters; A: % of fortification; %, B: Steaming time; min, C: volume of water; ml/kg of dry matter on responses with linear models (carbohydrates, fat, ash, soluble sugar, and minerals (P, K, Ca, Mg, Fe, and Zn).

Figure 4 presents two-dimensional RSM contour plots illustrating the effects of fortification level **(A)**, steaming time **(B)**, and water volume **(C)** on 10 nutritional and mineral responses, described by linear models. Fortification level emerged as the overwhelmingly dominant factor across all responses, while steaming time and water volume exerted negligible to no significant effects on most parameters. Fat, ash, and soluble sugar contents increased markedly with fortification level. Potassium and iron showed the greatest absolute enrichment, with K increasing nearly 5-fold and Fe approximately 4.6-fold across the fortification range, underscoring the high mineral density of the fortification ingredient. Phosphorus, calcium, and magnesium also increased positively with fortification, though over narrower ranges, contributing to improved overall micronutrient profiles. This finding indicates that quinoa is a richer source of lipids, minerals, and soluble sugars than rice. The near-vertical orientation of contour lines across most plots confirms the dominant linear role of fortification and the minimal contribution of processing parameters to compositional variation. Zinc was the only response plotted against steaming time and water volume, exhibiting only marginal variation within a narrow range, suggesting its concentration is primarily composition-dependent rather than process-dependent. The parallel contour patterns across all mineral plots indicate no significant interaction between fortification level and steaming time, simplifying the optimization framework.

Overall, fortification level emerged as the most critical factor driving techno-functional and nutritional properties, while steaming time and water volume played complementary modulating roles. The interactions among factors highlight the necessity of a multivariable optimization approach rather than a single-factor adjustment. These findings provide a reliable empirical basis for identifying optimal processing conditions that simultaneously maximize the functional and nutritional quality of the fortified product.

#### 3.2.3 Optimization of the variables and validation of the model

Numerical optimization was performed using the desirability function approach implemented in Design-Expert software to identify processing conditions that provide a balanced compromise among multiple quality attributes. The optimization aimed to maximize nutritional quality (fat, ash, TPC, P, K, Ca, Mg, Fe, and Zn), enhance techno-functional performance (OHC, SC, and WHC), and minimize undesirable factors (moisture and soluble sugar contents).

For the validation of the model, quinoa fortification was set at 100% and steaming time and water volume were targeted at their central values (20 min and 400 ml/kg, respectively). The optimization goals, constraints, and ranges applied to all factors and response variables are summarized in (Table 6).

**Table 6:** Optimization criteria for gluten-free quinoa–rice couscous processing.

Name	Goal	Importance	Predicted	Observed	Std dev	SE pred	95% PI low	95% PI high
WHC	maxi	3	3.21	3.29	0.05	0.09	3.00	3.42
OHC	maxi	3	0.82	0.81	0.04	0.05	0.71	0.93
SC	maxi	3	6.31	6.5	0.12	0.14	5.98	6.64
Moisture	mini	3	13.87	13.10	0.36	0.42	12.89	14.85
Carbohydrates	maxi	3	72.04	72.81	0.86	0.94	70.02	74.06
Fat	maxi	3	1.40	1.56	0.13	0.14	1.09	1.70
Ash	maxi	3	1.39	1.62	0.17	0.19	0.98	1.79
Soluble Sugar	mini	3	9.64	7.82	1.30	1.41	6.59	12.69
TPC	maxi	3	0.48	0.44	0.02	0.02	0.43	0.54
P	maxi	3	0.14	0.14	0.01	0.01	0.13	0.16
K	maxi	3	0.44	0.44	0.01	0.01	0.42	0.45
Ca	maxi	3	0.04	0.04	0.00	0.00	0.04	0.04
Mg	maxi	3	0.07	0.07	0.00	0.00	0.06	0.08
Fe	maxi	3	31.24	31.26	3.69	4.01	22.57	39.90
Zn	maxi	3	13.21	13.01	0.27	0.30	12.58	13.85

PI, prediction interval. Experimental validation performed in triplicate (n = 3) under optimized conditions: 100% quinoa fortification, 20 min steaming time, 400 ml/kg water volume. Desirability = 0.72.

The optimization procedure yielded a composite desirability value of 0.72, indicating a satisfactory compromise among the defined objectives. This moderate desirability value reflects the inherent trade-off between maximizing nutritional attributes and minimizing undesirable factors. The predicted response values corresponding to the optimized conditions are presented in Table 6.

To validate the predictive accuracy of the fitted models, experimental verification was conducted in triplicate under the optimized processing conditions (100% quinoa fortification, 20 min steaming time, and 400 ml/kg water volume). As shown in Table 6, the experimentally observed values for all validated responses were within the corresponding 95% prediction intervals. The close agreement between predicted and experimental values confirms the adequacy of the regression models for describing the system's behavior within the studied experimental domain.

### 3.3 Effect of quinoa fortification on nutritional composition of gluten-free couscous

**Figure 5:**
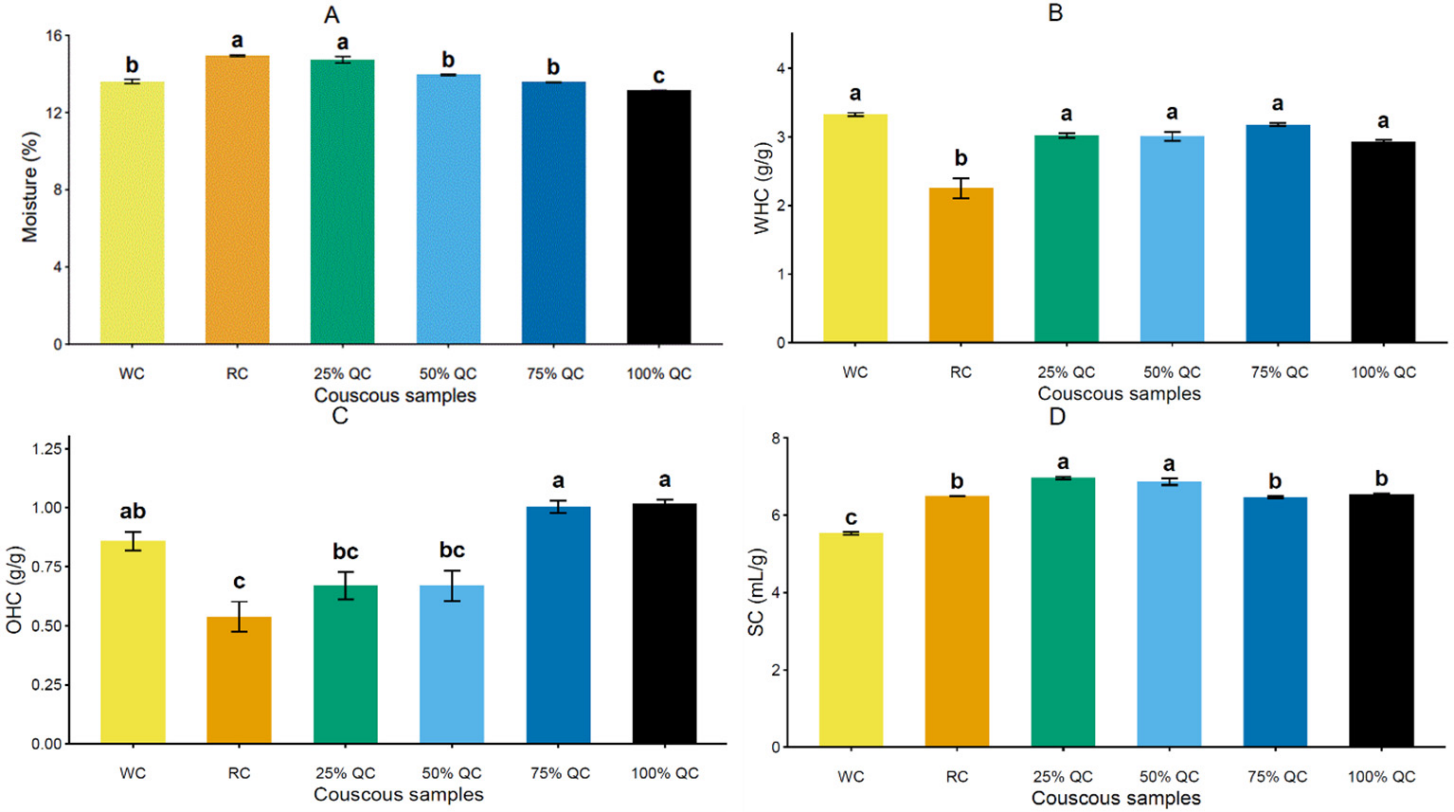
Variation of techno-functional properties: moisture **(A)**, WHC **(B)**, OHC **(C)**, and SC **(D)** of couscous samples.

**Figure 6:**
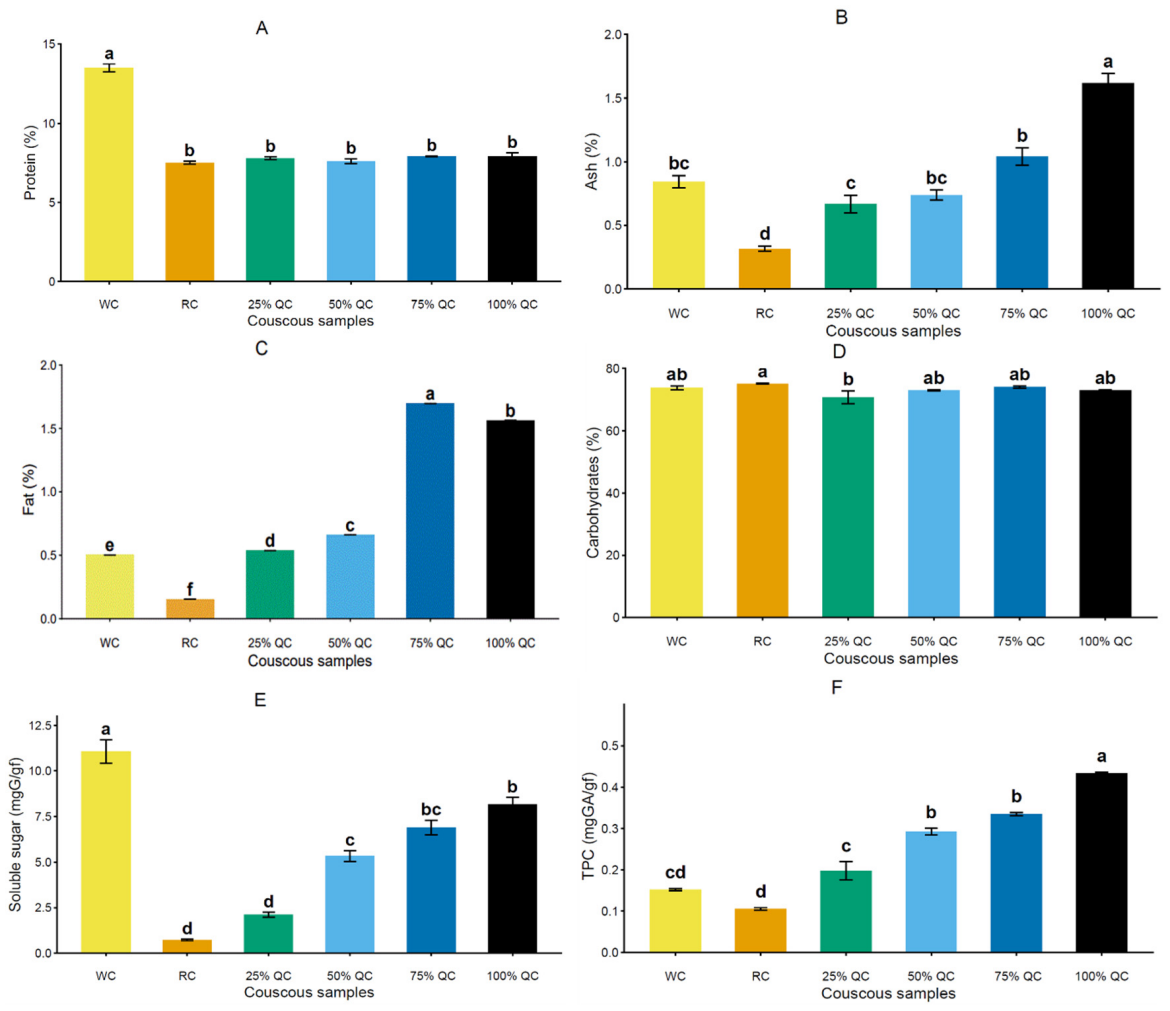
Effect of quinoa fortification percentage on proximate composition and bioactive compounds in gluten-free couscous: protein **(A)**, ash **(B)**, fat **(C)**, carbohydrates **(D)**, soluble sugar **(E)**, and TPC **(F)**.

**Figure 7:**
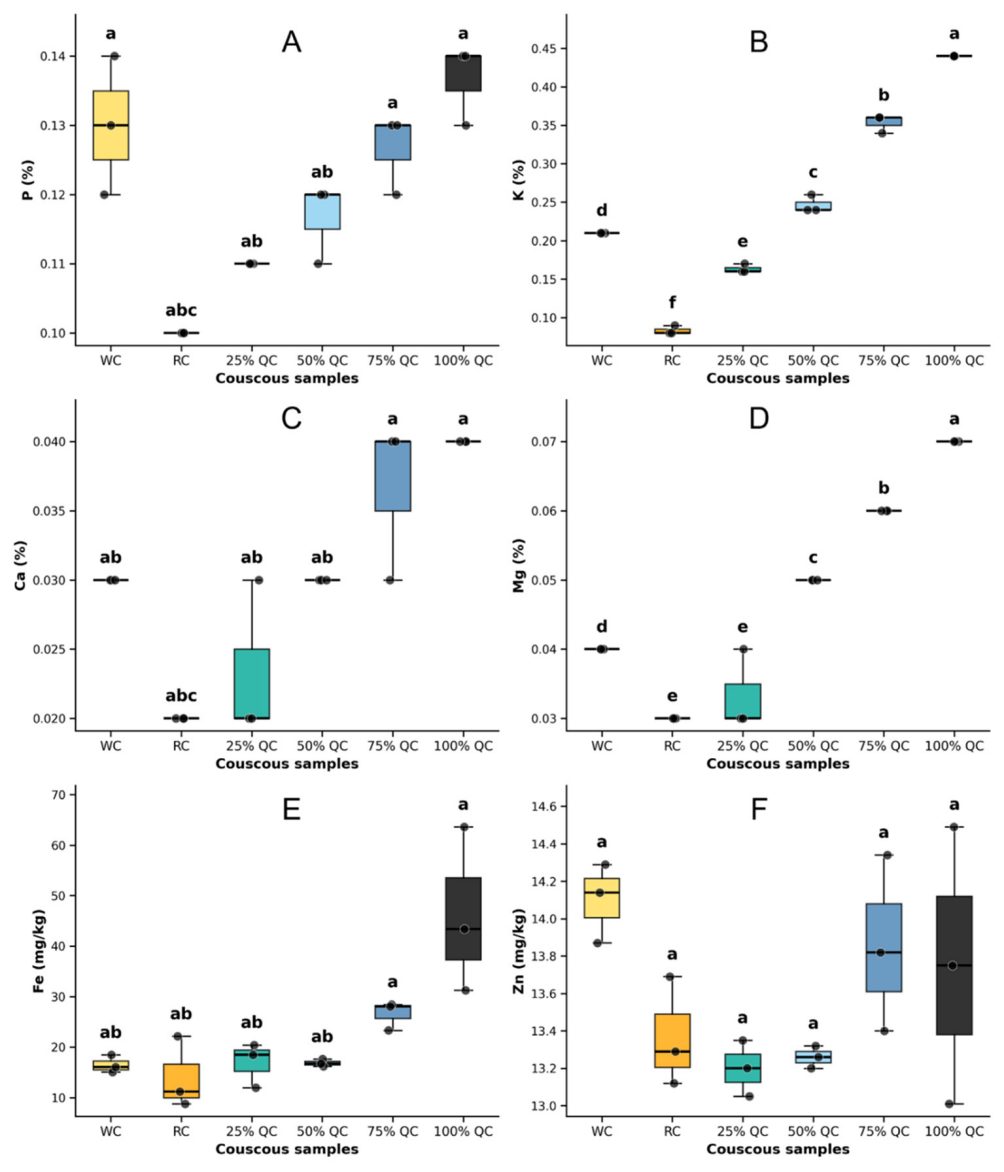
Variation of mineral content [Posphorus **(A)**, potassium **(B)**, calcium **(C)**, magnesium **(D)**, iron **(E)**, and zinc **(F)**] through couscous formulas.

**Figure 8:**
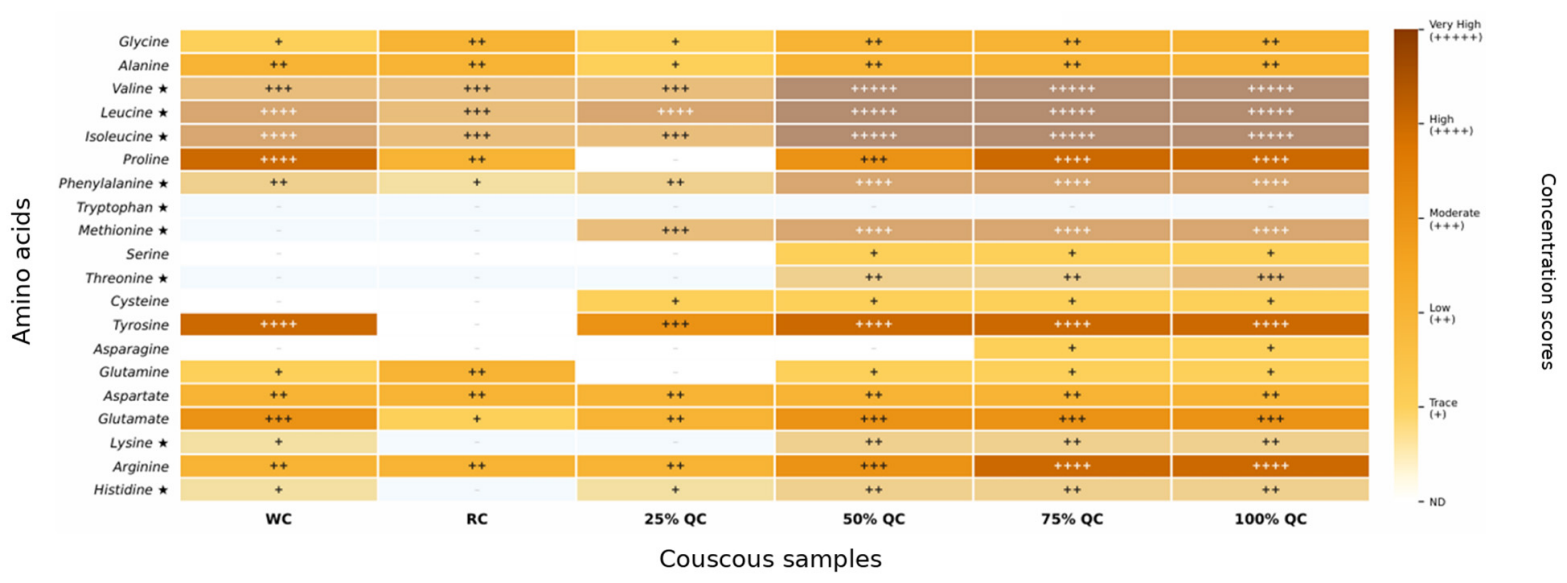
Amino acid detection score Heatmap of couscous samples. * Essential amino acids

**Figure 9:**
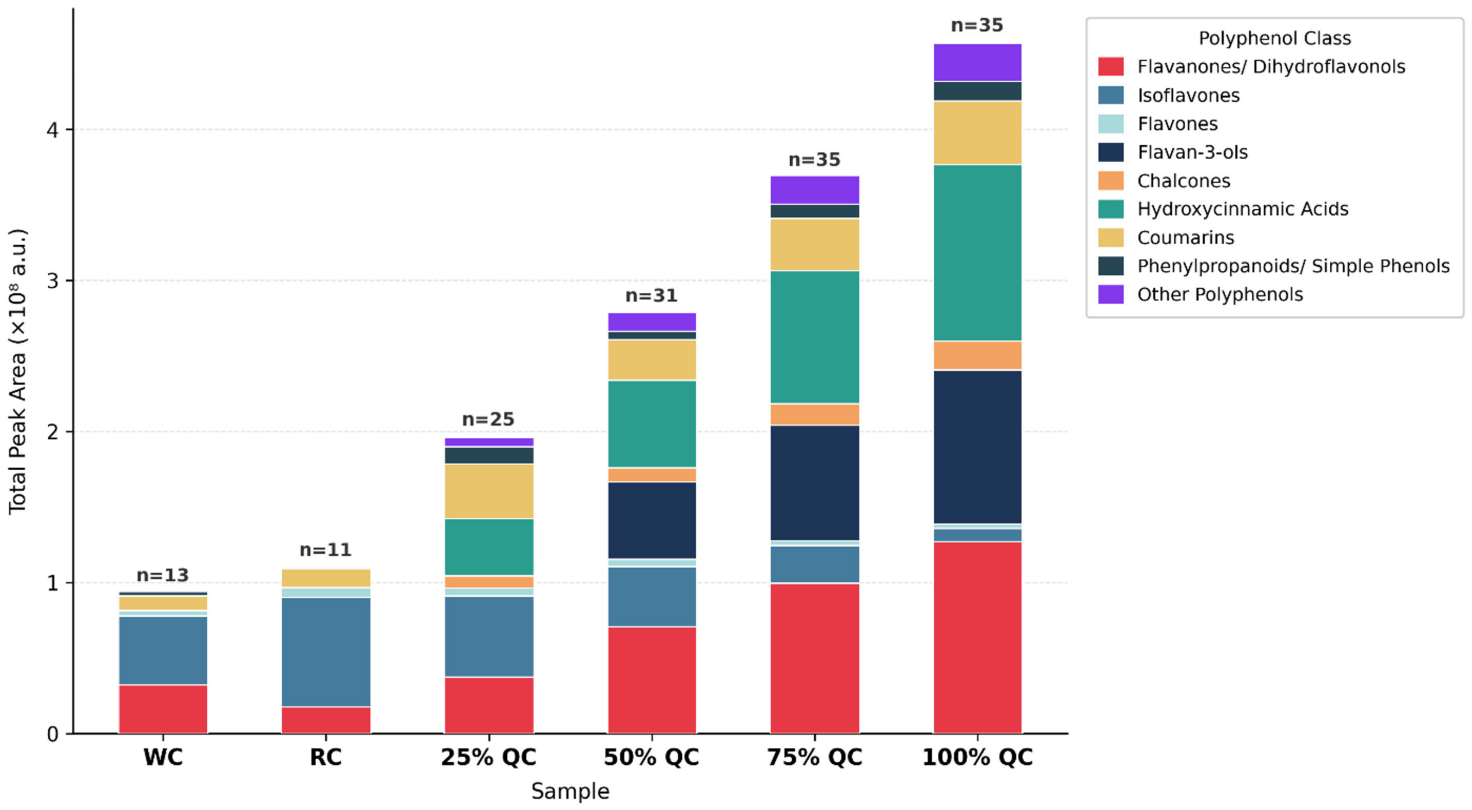
Comparative polyphenol profile by chemical class in couscous formulas.

**Figure 10:**
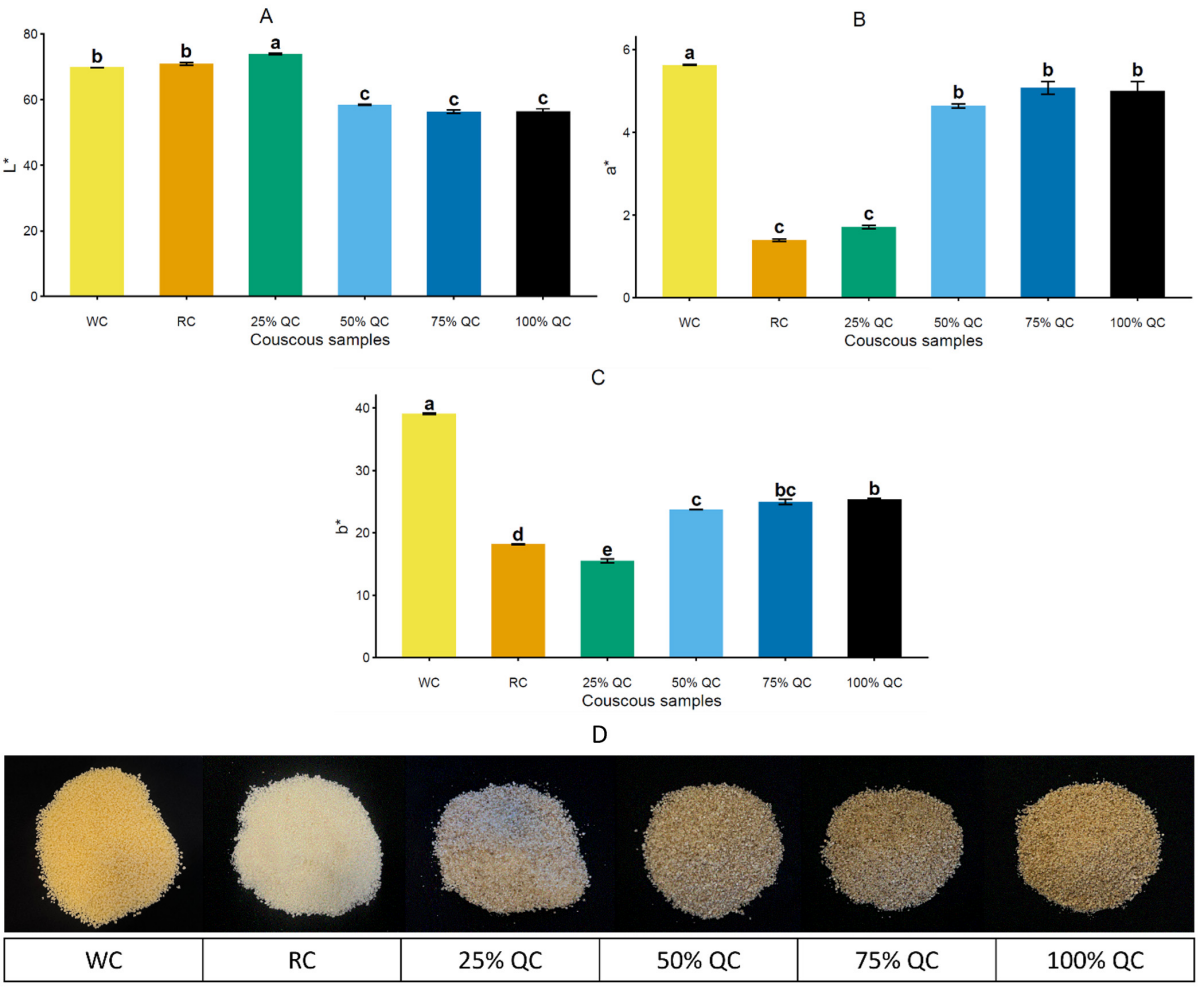
Variation of color parameters: L*(A), a*(B), b*(C), and visual appearance **(D)** of couscous formulas.

Based on the optimization results, which identified quinoa fortification percentage as the most influential factor, a detailed nutritional characterization of gluten-free couscous was conducted across five fortification levels (RC = 0% QC, 25% QC, 50% QC, 75% QC, and 100% QC) in comparison with commercial durum wheat-based couscous (WC). Steaming time and water volume were held constant at 20 min and 400 ml/kg, respectively. The results are presented in (Figures 5–10).

#### 3.3.1 Techno-functional properties

Figure 5 illustrates the effect of quinoa fortification percentage on moisture content **(A)**, water holding capacity (WHC, B), swelling capacity (SC, C), and oil holding capacity (OHC, D). The ANOVA results indicated a highly significant effect of fortification percentage on all techno-functional properties (*P* < 0.001).

Moisture content decreased significantly with increasing quinoa fortification level, from 15.01% at RC to 13.10% at 100% QC, indicating that quinoa incorporation reduces moisture retention in the couscous matrix. For WHC, quinoa-based couscous showed higher values; the presence of quinoa allowed couscous to hold more than threefold water compared to rice couscous, which held twofold water (2.00 g/g), with no significant difference from wheat couscous, which reached the maximum value of 3.33 g/g. Similarly, OHC increased with the presence of quinoa in the formulas, reaching a maximum of 1.02 g/g in 100% QC, while rice couscous exhibited the lowest OHC (0.54 g/g). For SC, no significant differences were observed among the RC, 75% QC, and 100% QC (6.50, 6.47, and 6.53 ml/g, respectively). In contrast, intermediate levels of quinoa fortification (25% QC and 50% QC) showed higher values (6.97 and 6.87 ml/g, respectively) than WC, which has a weak swelling capacity.

#### 3.3.2 Variation of nutrients and bioactive compounds

Figure 6 presents the variation of protein, ash, fat, carbohydrates, soluble sugar, and total phenolic content (TPC). Significant positive correlations were observed between the presence of quinoa semolina in the couscous formula and ash, fat, soluble sugar, and TPC contents.

The results show no significant difference in protein content across all fortification levels, while wheat couscous showed a higher protein content, which is heavily dependent on ingredient composition. Similarly, carbohydrates show no significant variation across all couscous samples. Regarding ash and fat content, a positive correlation between these compounds and quinoa fortification levels was observed, with levels increasing from 0.32% and 0.15% to 1.62% and 1.56%, respectively, across RC and 100% QC. Soluble sugar content also increased significantly with quinoa fortification, reaching 9.64 mg G/g dry matter at 100% QC. TPC showed a highly significant response to fortification (*P* = 5.33 × 10^−14^), confirming quinoa as the primary contributor of phenolic compounds in the couscous formulations.

#### 3.3.3 Variation of macro and micro–elements across formulated couscous samples

The box plot analysis (Figure 7) reveals distinct patterns in mineral content across six couscous formulations (WC, RC, 25% QC, 50% QC, 75% QC, and 100% QC). The one-way ANOVA demonstrated significant differences in mineral content among couscous formulations for P, K, Ca, Mg, and Fe (all *p* < 0.002). Tukey HSD *post hoc* tests revealed that quinoa enrichment progressively increased the content of P, K, Ca, Mg, and Fe. Quinoa enrichment consistently increased P content, with 100% QC showing 37% higher P than RC. The statistical groupings indicate that quinoa fortification percentages ≥75% achieve P levels comparable to wheat couscous (Figure 7A). Potassium showed the most dramatic variation (*F* = 903.7, *p* < 0.001), correlated with quinoa percentage, with values rising more than fivefold from 0.08% in rice couscous to 0.44% in 100% QC, demonstrating the high potassium content in quinoa. K content increases proportionately with each increase in quinoa %; no plateau effect is seen (Figure 7B).

Regarding calcium content (Figure 7C), a 2-fold increase was observed from RC (0.020%) to 100% QC (0.040%), with significant richness in couscous formulations with high quinoa fortification levels (75% and 100%) compared to wheat couscous. Magnesium has one of the clearest dose-response relationships. Each formulation is statistically distinct or forms a group with only adjacent treatments (e.g., RC and 25% QC), indicating precise control of Mg content through quinoa blending ratios (Figure 7D). Iron content increased approximately threefold, from 14.03 mg/kg in RC to 46.09 mg/kg in 100% QC (Figure 7E). In contrast, zinc content remained statistically similar across all couscous formulations (*P* = 0.082), suggesting similar Zn content in the wheat, rice, and quinoa ingredients (Figure 7F).

#### 3.3.4 Amino acids variation

The putative amino acid profiling of couscous samples by LC-MS/MS revealed marked differences in detection breadth and relative abundance between wheat couscous (WC), rice couscous (RC), and quinoa-based couscous. The QC gradient detected the highest number of amino acids (18–19/20 at ≥50% QC), compared to 14/20 in WC and only 11/20 in RC, indicating that integrating quinoa in couscous supports the amino acid diversity of couscous. Total concentration scores followed a clear dose-dependent pattern across QC fortification levels (34, 51, 54, 55 from 25% to 100% QC, respectively), confirming analytical linearity. The heatmap analysis (Figure 8) revealed the distribution of amino acid-related compounds across couscous samples. Branched-chain amino acids (BCAAs: leucine, isoleucine, valine) attained the highest scores (++++ to +++++) in all samples, with QC reaching very high (+++++) levels from 50% onwards. Proline and tyrosine were notably enriched in WC (++++ each) but dramatically reduced or absent in RC, suggesting their absence in used rice. Methionine and threonine were detected from the levels 25% and 50% QC, respectively, reflecting their enrichment in the QC matrix rather than in common couscous. Lysine, the primary limiting amino acid of wheat, was detected only at trace (+) in WC and was absent in RC, confirming the well-documented lysine deficiency of semolina-based products. Tryptophan remained undetected across all conditions. The radar chart (Supplementary Figure 2S) confirms the BCAA-dominant, nutritionally imbalanced profile of WC and RC relative to QC samples. Mean scores per detected amino acid were remarkably similar across samples (2.09–2.89), indicating that the qualitative gap between WC/RC and QC lies primarily in detection breadth rather than in the intensity of individually detected compounds.

#### 3.3.5 Comparative polyphenol profile by LC-MS

The LC-MS/MS polyphenol profiling of couscous samples putatively identified 44 compounds distributed across 9 chemical classes, with notable variations in both diversity and total abundance between wheat couscous (WC), rice couscous (RC), and the QC percentages (Figure 9 and Supplementary Table S2). The QC gradient consistently yielded the highest polyphenol diversity, with 35 compounds at 100% QC, versus only 13 in WC and 11 in RC, representing 4.9- and 4.1-fold enrichments, respectively. In WC and RC, isoflavones dominated the polyphenol profile (blue segment), accounting for approximately 65% of the total area in WC and over 75% in RC, accounting for the majority of the total peak area. Three isoflavones were detected (7-methoxy-2-methylisoflavone forms A and B, and 3′-methoxydihydroformononetin; rows 28–30 in Supplementary Table S2), with the methyl-isoflavone forms representing the most abundant class in both WC and RC. In contrast, the couscous quinoa-based shifted progressively toward hydroxycinnamic acids (teal) and flavan-3-ols (dark navy) as major contributors at ≥50% QC. All samples contained flavanones/dihydroflavonols (red), but the QC gradient showed a significant enrichment of these compounds. Chalcones, flavan-3-ols, and hydroxycinnamic acids were notably absent or below detection in both WC and RC, and they only appeared in the QC series starting at 25%, suggesting that quinoa is unique for these compounds. Coumarins (gold/yellow) were present at comparable levels in WC, RC, and throughout the QC gradient. Of the 7 chalcones tentatively annotated, 6 were undetected in WC and RC and appeared only in the QC samples; one chalcone 2′4′-dihydroxy-3,4-dimethoxychalcone) was detected at low level in all samples. Phenylpropanoids/simple phenols were also enriched in the quinoa formulations. Overall, these results demonstrate the limitations of wheat couscous in terms of polyphenols and provide compelling evidence for the nutritional justification of adding quinoa to the couscous formulation, considering that quinoa is characterized by a higher relative abundance of hydroxycinnamic acids (ferulic, caffeic), flavonoids (quercetin, kaempferol, rutin), and betacyanins, classes that are mostly lacking in both WC and RC.

#### 3.3.6 Effect of quinoa fortification on color

(Figures 10A, 10B, 10C) presents successively the color parameters (L^*^, a^*^, and b^*^) measured at each fortification level. Quinoa fortification significantly affected the lightness (L^*^) and redness (a^*^) of couscous formulations.

L^*^ values decreased progressively with increasing quinoa content, indicating that higher fortification levels result in darker products. The a^*^ parameter (redness–greenness) increased significantly (*P* = 2.77 × 10^−13^) from 0.945 in rice couscous to approximately 5.0 at 75% and 100% quinoa, with no significant difference between these two highest levels, and the maximum value was registered in wheat couscous (5.63). Similarly, the b^*^ parameter (yellowness–blueness) was high for WC, with borderline significance (*P* = 0.051) between the gluten-free couscous formulas.

The color transition is visually illustrated in Figure 10D, which shows the progressive change from white (0% quinoa) to brown as quinoa content increases. The color stabilized between 75% and 100% fortification, suggesting saturation of pigment contribution at higher quinoa levels.

## 4 Discussion

The nutritional quality of couscous ingredients was evaluated based on the variation of protein, carbohydrates, fat, ash, moisture, mineral profile, saponin content, total phenolic content, color measurements, and techno-functional properties. The quinoa semolina showed high levels of most nutrients, including fat, ash, and minerals. Several studies reported that quinoa is gluten-free and contains a balanced nutrient profile, compared with other cereals like rice, corn, and barley (25, 27). Quinoa is characterized by its high protein content, including all essential amino acids; high dietary fiber; and low carbohydrate content (32, 33). In our case, a non-significant difference between rice and quinoa was registered for protein content, which contradicts the well-documented protein richness of whole quinoa grain (typically 12–17% on a dry weight basis) (11, 34). This apparent contradiction is resolved when the milling step is considered. In order to produce quinoa semolina, the outer saponin-containing layer must be polished, and then the perisperm fraction must be recovered by milling and sieving. This process eliminates a significant amount of the protein-rich germ (up to 70% of the total protein) (35). Similarly, rice polishing removes the protein-rich bran layer from rice grains before milling into flour. Consequently, the milled semolina and flour ingredients used in this study have comparable protein contents, which explains the lack of a significant fortification effect on couscous protein. The protein content results are in line with many other studies that reported a value of 5.9% and 7.2%. The milling process separates the embryo from the seed, which is a protein-rich component, and essentially retains the perisperm, which accounts for the low protein concentration (25, 35). This milling exhibited a saponin content of 0.53 mg OA/g, yielding a sweet quinoa ingredient, as the saponin content is below the dietary threshold (0.11%) (10, 11). Quinoa semolina also contains antioxidant compounds such as polyphenols; Pathan & Siddiqui (2022) reported that the total phenolic content in quinoa seeds ranged from 0.39 to 1.98 mg GAE g^−1^ dry weight, which is in accord with our finding. For the color measurements, the most dramatic differences between quinoa and rice are in lightness (L^*^) and yellowness (b^*^). Rice is much lighter and much less yellow than quinoa; this color can be associated with levels of phenolic compounds and pigments in quinoa compared to rice (16, 36). On the other hand, the high levels of the techno-functional properties of quinoa are likely due to structural and compositional differences among the raw materials (37). Integrating quinoa into traditional Mediterranean products like couscous is hypothesized to significantly improve their overall nutritional quality, given quinoa's exceptional richness in complete protein, dietary fiber, and essential micronutrients such as iron, potassium, and magnesium (34, 38).

Developing quinoa-based couscous involves significant obstacles. These include adjusting processes to preserve quinoa's nutritional characteristics and attractive texture and ensuring the finished product closely mimics the cooking behavior of conventional couscous. Response surface methodology (RSM) is an approach commonly used to optimize food processes because of its capacity to effectively model, examine, and enhance intricate processes with numerous variables (15). In our study, we used a D-optimal design in RSM to improve efficiency in the couscous formulation, minimize processing costs, and maintain the nutritional quality of the ingredients. The effects of three factors were studied: the percentage of quinoa fortification (A, %), the steaming time (B, min), and the volume of water (C, ml/Kg of ingredient). The responses included three techno-functional properties (OHC, WHC, SC), protein, fat, ash, carbohydrates, moisture, minerals (P, K, Ca, Mg, Fe, Zn), and total phenolic content (TPC). To ensure the models for these responses are correct, a sufficiency check was performed. This step is important because an accurate, precise mathematical model can improve the real process. Conversely, an incorrect model can lead to poor or misleading results (39). Based on ANOVA results, regression, and residual distributions, the models are statistically accurate and appropriate for optimization and prediction across all studied responses. The couscous processing was optimized utilizing these responses, which had verified and trustworthy models.

The evaluation of factor effects on couscous nutrients, based on mathematical equations, showed a positive impact of the percentage of quinoa fortification on most of the studied responses. This can be justified by the fact that incorporating quinoa into food products results in substantial improvements in their nutritional profiles. Quinoa is rich in high-quality nutrients and bioactive compounds, making it a powerful ingredient for boosting the health value of various foods (40, 41). Steaming time positively affects swelling capacity and negatively affects OHC and WHC, without any impact on TPC, minerals, or other nutrients. This pattern is consistent with the broader literature, in which atmospheric steaming has been shown to preserve minerals, phenolics, and antioxidant activity in both pseudocereals and rice more effectively than boiling, primarily because soluble nutrients do not leach into the cooking water (25, 42, 43). Within the steaming process itself, the duration of treatment can modulate nutrient retention; Zohoun et al. (2018) found that variations in steaming times for precooking rice can enhance the nutritional quality of certain rice varieties, including proteins, potassium, magnesium, and lipids, while a non-significant effect was observed for calcium and starch (44). The volume of water used during couscous processing is a critical parameter that influences not only the product's physical properties but also its nutritional quality. Water addition affects nutrient retention and the leaching of soluble nutrients (TPC in our case). Research shows that both the amount and temperature of water during mixing, agglomeration, and cooking stages can alter the content of protein, fiber, minerals, and bioactive compounds in couscous, as well as its functional and sensory properties (45).

Furthermore, response surface contours are essential tools for identifying optimal processing conditions and visualizing the factor interactions (46). In our study, this tool provided optimal conditions for preserving the maximum nutrients in couscous formulas. Overall, the adaptation of the factors, volume of water, and the steaming time depends on the ingredients used.

The limited influence of steaming time and water volume on nutritional composition likely reflects the thermal stability of the target nutrients under the mild processing conditions tested (10–30 min atmospheric steaming, no direct water contact during cooking). Unlike boiling, where soluble nutrients leach into the cooking water, the steaming step in traditional couscous processing exposes granules only to vapor, which preserves minerals, phenolics, and protein integrity more effectively than hydrothermal treatments with direct water contact (25). This suggests that, for couscous-type products, the formulation decision, not the processing window, is the primary lever for nutritional quality, which simplifies product development by reducing the number of process variables that must be optimized in parallel. This contrasts with pasta and noodle systems, where water volume and cooking time materially affect protein and mineral retention and suggests that couscous-type products may be a more robust vehicle for nutrient fortification than boiled cereals (47).

The percentage of fortification has more strongly affected nutrient variation, masking the effects of the other processing parameters for most of the studied properties. Consequently, a comprehensive characterization of couscous formulas based on variation in this important factor is necessary to assess the nutritional quality of formulated couscous. A key aspect of this understanding involves techno-functional properties; the results showed a positive correlation between adding quinoa to couscous and enhancing these properties. In the same context, several studies have demonstrated that the percentage of quinoa in a food product directly affects its techno-functional properties, including water and oil absorption, foaming, swelling, emulsification, and gel formation (48). Adding more quinoa usually improves the nutritional value and some functional properties; this increase is due to quinoa's protein, lipid, and fiber content (20, 49, 50). In noodles, a higher quinoa percentage increases water and oil absorption and the swelling index, thereby improving hydration and texture (48).

Likewise, a positive impact of quinoa on nutrient levels was observed; this is consistent with previous findings showing that increasing quinoa content in food products leads to higher nutrient levels, making it an excellent source for food applications (19, 51). Similarly, polyphenol levels increased with increasing quinoa incorporation. For example, snacks made with 50% of quinoa flour showed the highest levels of free and bound polyphenols, reaching up to 14.16 mg GAE/g, as well as strong antioxidant activity. The polyphenol content can vary depending on quinoa variety, but increasing quinoa intake usually increases total phenolic content and enhances antioxidant properties (52). Quinoa also contains natural sugars such as sucrose, glucose, and fructose. As more quinoa is added to a food product, the sugar content increases slightly. Still, it stays low compared to other main nutrients (19).

Closely, quinoa fortification plays a dominant role in determining macro- and microelement levels in couscous formulas. Phosphorus, potassium, calcium, magnesium, and iron content were all considerably increased by quinoa enrichment in couscous, with potassium exhibiting the most notable 5.3-fold increase and zinc remaining constant among formulations. Correspondingly, several studies demonstrated that quinoa is a good source of important minerals such as potassium, magnesium, calcium, and iron. Increasing the amount of quinoa in a product usually raises its total mineral content (25, 53). The 100% QC provides more K than bananas (0.36%) and potatoes (0.42%) and is comparable to leafy green vegetables like broccoli, kale, and spinach (0.4–0.7%) (54–57). In terms of magnesium, which is associated with various physiological functions, quinoa-based couscous offers a significant amount of magnesium compared with conventional wheat couscous; 100% QC provides 17–23% of daily Mg needs per 100 g serving, providing a meaningful contribution to recommended daily intake (17, 58). A similar trend was observed for iron, 75–100% quinoa formulations can provide between 26 and 62% of the reference daily intake reported for children, depending on the age range considered. The high iron and magnesium contents observed may be nutritionally relevant given the widespread concern over insufficient dietary intake of these minerals, but their actual contribution to mineral status cannot be inferred from the present data, as plant-derived mineral bioavailability was not assessed (59).

The semi-quantitative amino acid analysis suggests that quinoa-based couscous is a nutritionally dense source of amino acids. Quinoa couscous showed a notable abundance of branched-chain amino acids (BCAAs), especially Val, Leu, and Ile (11, 60). Lysine is the first limiting amino acid in cereal proteins, which directly impacts the protein quality score of couscous as a sole protein source. This limitation has serious implications for public health because wheat and rice together are the main sources of protein for many people, especially in North Africa, the Middle East, and Asia, where plant-based diets are common and animal protein consumption is low (61, 62). In this regard, the incorporation of quinoa into couscous-like staple food in these regions provides a great solution to enhance essential amino acid' richness, cognizant of the similarity of quinoa protein to casein (34). Quinoa protein can provide approximately 180–338% of the nine essential amino acids that are recommended in protein sources for adult nutrition, according to FAO, WHO, and UNU (34). Tryptophan was not detected in any of the six samples, including the QC samples spiked with the analyte. This absence may reflect either degradation during processing (e.g., through Maillard-type reactions involving the indole side chain) or the well-documented low ionization efficiency of tryptophan in ESI under standard reverse-phase LC–MS conditions, which would require the optimization of isolation and analysis method in future work (63).

The polyphenol profiling of wheat couscous (WC), rice couscous (RC), and quinoa couscous (QC) revealed several analytically and nutritionally significant findings. The QC presents a markedly richer and more diverse phenolic compounds compared to WC and RC, both in terms of compound count (35 compounds at ≥75% QC vs. 13 in WC and 11 in RC) and total integrated peak area (~4.9-fold higher in 100% QC than WC). Beyond this overall enrichment, QC is characterized by the exclusive presence of flavan-3-ols, chalcones, and hydroxycinnamic acids that increase dose-proportionally across the QC gradient. These compounds were registered in different quinoa varieties, conferring a significantly higher estimated antioxidant capacity (11, 34, 64). The detection of dimethylcaffeic acid and ferulic acid in the quinoa formulations is particularly relevant given that ferulic acid and related hydroxycinnamates are among the polyphenols most consistently associated with the *in vitro* antioxidant capacity of quinoa (19, 64, 65). The single putatively identified flavan-3-ol (gallocatechin4′-methyl ether) followed the same pattern, increasing with quinoa fortification, while six of seven detected chalcones were quinoa-associated. In WC and RC, by contrast, the polyphenol profile was dominated by a small number of isoflavones, with no comparable diversification across compound classes. The following findings indicate that consumers who rely exclusively on conventional couscous as a dietary source of polyphenols have limited intrinsic antioxidant coverage. That reformulation with quinoa, alongside dietary diversification with legumes, vegetables, and whole fruits, represents a nutritionally meaningful strategy to improve the phenolic quality of this culturally central staple food (34). The use of untargeted LC-HRMS profiling to characterize couscous represents a methodological shift relative to prior studies of quinoa- or alternative-grain-enriched couscous, which have predominantly relied on targeted assays of total phenolic content or single-class spectrophotometric measurements. Untargeted high-resolution MS allows simultaneous detection of compounds across multiple chemical classes from a single injection, and at the analytical confidence level achievable here (MSI level 2, putative annotation by spectral library matching), it provides a compound-level fingerprint of the formulation that cannot be obtained from total-polyphenol or class-summed approaches alone (30, 66). Recent applications of this approach to quinoa and pseudocereal matrices have similarly revealed compound diversity that is invisible to single-assay measurements (67).

For couscous appearance, the percentage of quinoa in food products plays an important role in their color, which affects product appearance and consumer acceptance. Higher quinoa content generally lowers the L^*^ value, resulting in darker products due to browning reactions and natural pigments in quinoa. The addition of quinoa, especially levels above 50%, increases the browning index and reduces lightness (L^*^). Changes in a^*^ and b^*^ values depend on the type of product and processing method (52). Adding quinoa changes the intensity of the red-green coloration. For instance, quinoa had little effect on color in sausages but led to darker colors and more red/yellow tones in snacks and extruded products (68). Overall, increasing quinoa content introduces more of its naturally brown-hued bran and pigments into the light white rice, steadily darkening the couscous's appearance. The stabilization of color between 75% and 100% quinoa indicates that the couscous matrix has reached a point of pigment saturation, where additional quinoa no longer alters the visible color, as the dominant brown tone has been fully established. The progressive darkening observed at quinoa fortification levels above 50% has direct implications for consumer acceptance; traditional North African consumers, accustomed to the light yellow-gold appearance of wheat couscous, may initially perceive darker products as less appealing (14). Conversely, in health-oriented and gluten-free market segments, darker color is increasingly associated with whole-grain authenticity and higher nutritional value, which may favor acceptance (52). Confirming the balance between these two effects requires targeted sensory and consumer-acceptance studies, particularly for blended formulations.

Our study offers a methodology for formulating gluten-free quinoa-based couscous using mathematical models that predict process outcomes under various conditions, enabling the determination of optimal settings before conducting further experiments. This study provides a basis for the development of a reproducible production process that could be investigated and adapted to other quinoa varieties in future work. It can also be combined with other cereals or legumes while keeping the nutritional quality of the raw material. The production of quinoa-based couscous showed that the processing steps did not negatively affect its nutritional quality. It offered a new perspective on developing innovative recipes, especially using quinoa varieties rich in protein. The research also emphasized the importance of optimizing raw material processing, including soft saponin removal and semolina production, as well as conducting stability and functionality tests.

This study has several limitations that should be acknowledged. First, the D-optimal design, while efficient, was restricted to the studied three factors; additional parameters such as drying temperature, particle size distribution, and flour-to-semolina ratio may influence product quality and warrant investigation. Second, sensory evaluation and consumer acceptability testing were not conducted, yet these are critical for market viability given the color darkening observed at 50% or higher quinoa fortification. Third, polyphenol and amino acid identifications relied on spectral library matching without authentic standards for all compounds. In addition, *in vitro* bioaccessibility and *in vivo* bioavailability of the identified nutrients were not assessed, so the dietary contributions reported above represent nutrient content rather than absorbed nutrient delivery. As well, instrumental texture profile analysis (TPA) of cooked couscous was not performed in the present study; characterization of attributes such as hardness, cohesiveness, adhesiveness, springiness, and chewiness would complement the techno-functional measurements reported here and provide a more complete picture of product quality. Finally, the single quinoa variety assessed (Puno) limits the generalizability of the results; varietal effects on nutritional composition, mainly protein content, saponin level, phenolic composition, and seed color are known to vary substantially among quinoa varieties in addition to processing behavior, which remains to be explored.

As future directions, whole-sweet quinoa grain should be evaluated as an alternative to polished semolina, since it would retain the protein-rich germ fraction removed during milling while preserving the mineral and polyphenol advantages demonstrated here; this approach would require integration with established saponin-removal protocols to maintain the below-threshold saponin content observed in the present formulations. Second, dry-fractionated quinoa fractions enriched in protein and lipids may offer a middle path that preserves the granulometric properties needed for traditional couscous processing while improving nutritional density. Extending both approaches across multiple quinoa varieties would simultaneously address the milling-induced protein limitation and the single-variety scope of the present study.

## 5 Conclusion

The present study aims to develop a gluten-free couscous that highlights the health benefits of quinoa and offers a nutritionally enhanced substitute for individuals with gluten intolerance, broadening their food selection while satisfying dietary requirements, fusing innovation, flavor, and health to support an inclusive, well-balanced diet.

The optimization of the chosen parameters (quinoa fortification percentage, steaming time, and water volume) at adequate levels was performed using response surface methodology. Most nutritional responses were positively correlated with the percentage of quinoa fortification, while steaming time and water volume acted as secondary modulators for selected responses.

Therefore, a detailed characterization of couscous formulations was conducted to evaluate the effect of quinoa fortification level on the nutritional quality of gluten-free couscous. The results demonstrate that adding quinoa improves techno-functional qualities and raises nutrient levels, particularly polyphenol compounds; minerals such as potassium, iron, and magnesium, in addition to amino acid diversity. These improvements make quinoa couscous a nutritionally relevant, nutrient-dense alternative to conventional wheat couscous, particularly within gluten-free and health-oriented dietary patterns.

This research successfully established a reproducible process for producing quinoa-based couscous, demonstrating that the processing steps preserve its high nutritional quality. The study provides a basis for using diverse ingredients, particularly protein-rich ones, and for creating innovative blends with other cereals or legumes. Further research on bioavailability, consumer acceptance, and health outcomes will strengthen the evidence base for its promotion as a functional food.

## Data Availability

The raw data supporting the conclusions of this article will be made available by the authors, without undue reservation.
