# Supplementary material for: Process optimization and nutritional profiling of gluten-free quinoa–rice couscous: amino acid and polyphenol characterization
Source: Front Nutr. 2026 Jul 10;13:1861043. doi: 10.3389/fnut.2026.1861043 (PMC13398055; doi:10.3389/fnut.2026.1861043)
Supplement: Supplementary file 1 [file Table_1.docx]

**Process Optimization and Nutritional Profiling of Gluten-Free Quinoa–Rice Couscous: Amino Acid and Polyphenol Characterization**

**Khadija El Hazzam^1†^, Kawtar Bettat^1,2†^, Mohamed Louay Metougui^3^, Didier Bazile^4,5,6^, Manal Mhada^1^***

^1^AgroBioSciences Department (AgBS), College of Agriculture and Environmental Science (CAES), Mohammed VI Polytechnic University, Benguerir 43150, Morocco

^2^Faculty of Sciences and Techniques, Hassan II University, Mohammedia, 28800, Morocco

^3^Agricultural Innovation and Technology Transfer Center (AITTC), College of Agriculture and Environmental Science (CAES), Mohammed VI Polytechnic University, Benguerir 43150, Morocco

^4^QUALINOA SAS de l’ESS, F-34270 Claret, France

^5^CIRAD, UMR SENS, F-34398 Montpellier, France

^6^UMR SENS, CIRAD, IRD, University Paul Valery Montpellier 3, University Montpellier, F-34090 Montpellier, France

^†^ The authors contribute similarly to the preparation of the manuscript.

**^*^ Corresponding author**:

Khadija El Hazzam

[Khadija.elhazzam@um6p.ma](mailto:Khadija.elhazzam@um6p.ma)

**Appendix A. Supplementary data**

**Table 1S**

Experimental design run

| Run | Factor 1: X_1_  percentage of quinoa fortification (%) | Factor 2: X_2_  Steaming time  (min) | Factor 3: X_3_  Volume of water (mL/kg) |
| --- | --- | --- | --- |
| 1 | 50 | 20 | 400 |
| 2 | 100 | 30 | 400 |
| 3 | 50 | 20 | 400 |
| 4 | 50 | 10 | 350 |
| 5 | 0 | 20 | 450 |
| 6 | 50 | 30 | 350 |
| 7 | 50 | 20 | 400 |
| 8 | 100 | 20 | 450 |
| 9 | 50 | 20 | 400 |
| 10 | 50 | 10 | 450 |
| 11 | 50 | 30 | 450 |
| 12 | 50 | 20 | 400 |
| 13 | 0 | 30 | 400 |
| 14 | 100 | 10 | 400 |
| 15 | 0 | 20 | 350 |
| 16 | 100 | 20 | 350 |
| 17 | 0 | 10 | 400 |


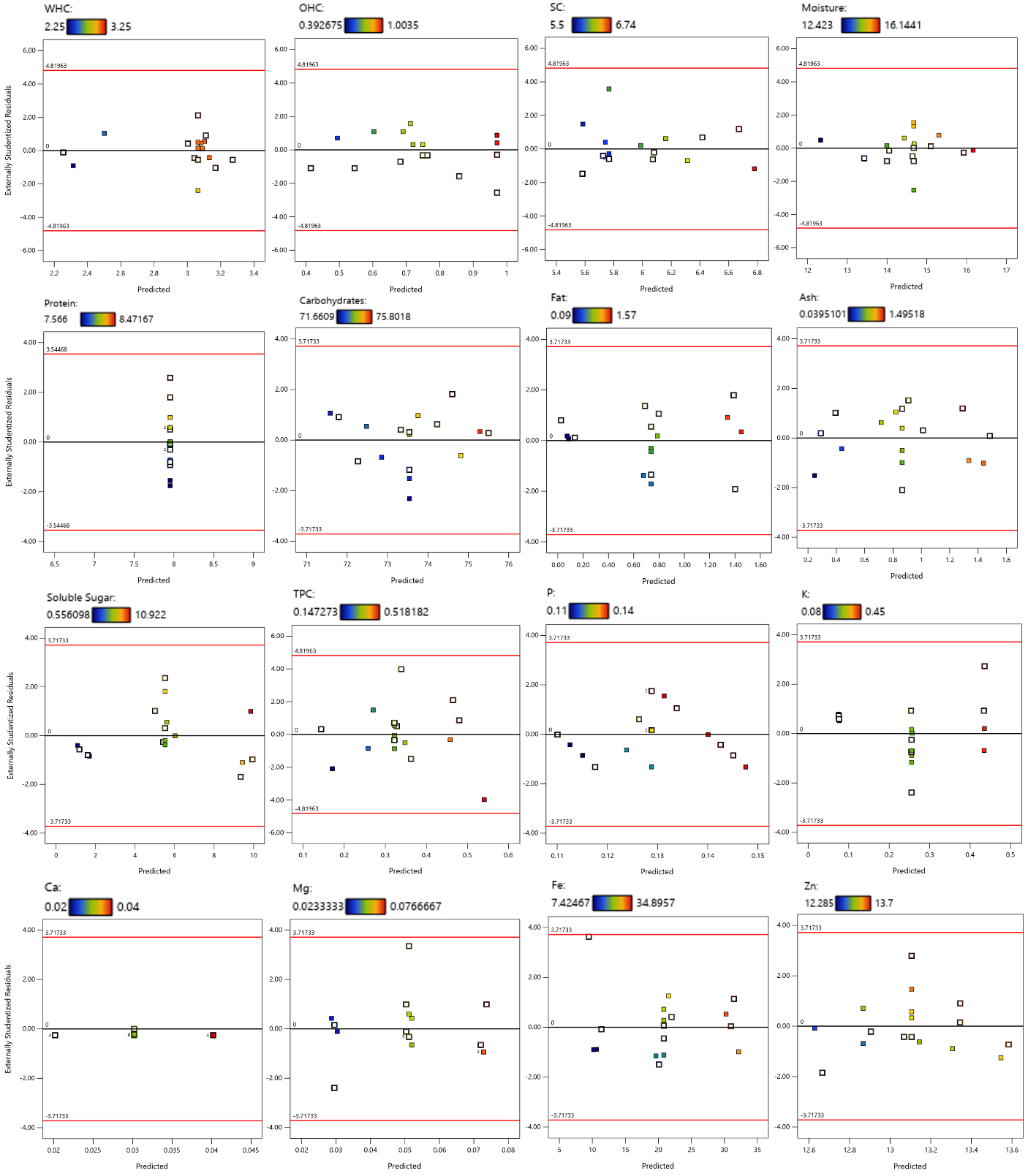


**Figure 1S.** Analysis of Residuals versus. Predicted values for proximate composition (Protein (%), fat (%), carbohydrates (%), ash (%)), techno-functional properties (OHC, WHC, SC), TPC (mg GA/g dry matter), Soluble Sugar (mg G/g dry matter), and minerals ((P, K, Ca, Mg, Fe, and Zn) as responses.


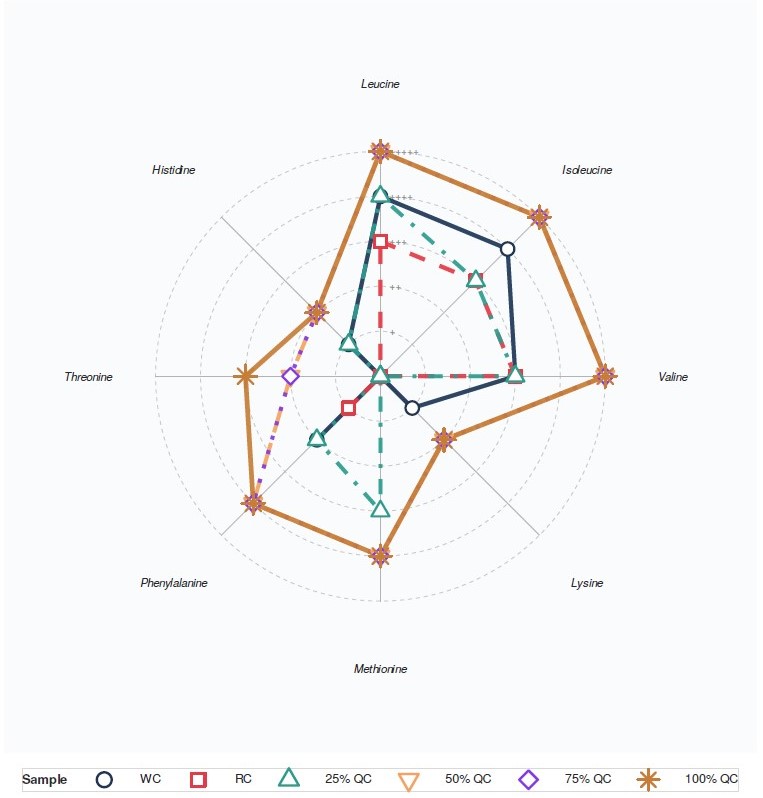


**Figure 2S.** Radar of couscous samples essential amino acid profile

**Table 2S**

Complete polyphenol profile of couscous samples detected by untargeted LC-HRMS/MS

|  | Compound Name | Chemical Class | Mol. Formula | RT (min) | m/z | Precursor Ion | Mode | mzCloud Score (%) | WC | RC | 25% QC | 50% QC | 75% QC | 100% QC |
| --- | --- | --- | --- | --- | --- | --- | --- | --- | --- | --- | --- | --- | --- | --- |
| 1 | *2',4'-Dihydroxy-3,4-dimethoxychalcone* | **Chalcones** | C₁₇H₁₆O₅ | 7.88 | 299.09282 | [M−H]⁻ | − | N/A | **+** | **+** | **++** | **+** | **+** | **+** |
| 2 | *Trihydroxychalcone* | **Chalcones** | C₁₅H₁₂O₄ | 6.68 | 255.06655 | [M−H]⁻ | − | 26.5 | – | – | **+** | **+** | **+** | **+** |
| 3 | *2',3',4',6'-Tetramethoxychalcone* | **Chalcones** | C₁₉H₂₀O₅ | 7.10 | 327.12418 | [M−H]⁻ | − | N/A | – | – | **+** | **+** | **+** | **+** |
| 4 | *Isoneobavachalcone* | **Chalcones** | C₁₇H₁₄O₅ | 6.22 | 297.07722 | [M−H]⁻ | − | N/A | – | – | – | **+** | **+** | **+** |
| 5 | *Phloretin* | **Chalcones** | C₁₅H₁₄O₅ | 4.53 | 273.07718 | [M−H]⁻ | − | 8.7 | – | – | – | **+** | **+** | **+** |
| 6 | *4'-Hydroxychalcone* | **Chalcones** | C₁₅H₁₂O₂ | 8.67 | 223.07673 | [M−H]⁻ | − | 8.8 | – | – | **+** | – | – | – |
| 7 | *Pongachalcone II* | **Chalcones** | C₂₁H₂₀O₅ | 6.67 | 351.12344 | [M−H]⁻ | − | N/A | – | – | **+** | – | – | – |
| 8 | *Swietenocoumarin F* | **Coumarins** | C₁₇H₁₈O₆ | 2.30 | 363.10815 | [M−H]⁻ | − | 55.2 | – | – | **+** | **++** | **++** | **++** |
| 9 | *Mutisifurocoumarin* | **Coumarins** | C₁₆H₁₀O₅ | 8.40 | 281.04585 | [M−H]⁻ | − | N/A | **+** | **++** | **++** | **+** | **+** | **+** |
| 10 | *6-Methoxy-8-hydroxyisocoumarin-3-carboxylic acid* | **Coumarins** | C₁₁H₈O₆ | 4.85 | 235.02502 | [M−H]⁻ | − | N/A | – | – | – | **+** | **+** | **+** |
| 11 | *3,4,7-Trihydroxy-6-methylcoumarin* | **Coumarins** | C₁₀H₈O₅ | 3.59 | 207.03007 | [M−H]⁻ | − | N/A | – | – | **+** | – | – | – |
| 12 | *Gallocatechin 4'-methyl ether* | **Flavan-3-ols** | C₁₆H₁₆O₇ | 3.25 | 301.07207 | [M−H]⁻ | − | N/A | – | – | – | **++** | **++** | **+++** |
| 13 | *Hesperetin (form A)* | **Flavanones/Dihydroflavonols** | C₁₆H₁₄O₆ | 2.55 | 301.07190 | [M−H]⁻ | − | N/A | **++** | **+** | **++** | **++** | **++** | **++** |
| 14 | *Hesperetin (form B)* | **Flavanones/Dihydroflavonols** | C₁₆H₁₄O₆ | 3.27 | 303.08612 | [M+H]⁺ | + | N/A | – | – | – | **+** | **++** | **++** |
| 15 | *Hesperetin (form C)* | **Flavanones/Dihydroflavonols** | C₁₆H₁₄O₆ | 6.13 | 257.08225 | [M−H]⁻ | − | N/A | **++** | **+** | **++** | **+** | **++** | **++** |
| 16 | *6-Methoxytaxifolin* | **Flavanones/Dihydroflavonols** | C₁₆H₁₄O₇ | 3.39 | 317.06700 | [M−H]⁻ | − | N/A | – | – | – | **+** | **+** | **++** |
| 17 | *4,2',4'-Trihydroxy-3-methoxydihydrochalcone* | **Flavanones/Dihydroflavonols** | C₁₆H₁₆O₅ | 5.92 | 287.09283 | [M±H]⁺/⁻ | ± | N/A | – | – | – | **+** | **+** | **++** |
| 18 | *(-)-5,7-Dihydroxy-3-(4-hydroxybenzyl)-4-chromanone* | **Flavanones/Dihydroflavonols** | C₁₆H₁₄O₅ | 5.63 | 285.07718 | [M−H]⁻ | − | N/A | – | – | **+** | **+** | **+** | **+** |
| 19 | *3,5,7,2'-Tetrahydroxy-5'-methoxyflavanone* | **Flavanones/Dihydroflavonols** | C₁₆H₁₄O₇ | 5.44 | 317.06714 | [M−H]⁻ | − | N/A | – | – | – | – | **+** | **+** |
| 20 | *3',4'-Dihydroxy-7-methoxy-8-prenylisoflavanone* | **Flavanones/Dihydroflavonols** | C₂₃H₂₂O₆ | 7.53 | 393.13415 | [M±H]⁺/⁻ | ± | N/A | **+** | – | – | **+** | **+** | **+** |
| 21 | *5,7-Dimethoxy-6-C-methylflavone (form A)* | **Flavones** | C₁₈H₁₆O₄ | 6.35 | 295.09795 | [M±H]⁺/⁻ | ± | N/A | **+** | – | **+** | **+** | **+** | **+** |
| 22 | *7,8-Dimethoxyflavone* | **Flavones** | C₁₇H₁₄O₄ | 7.01 | 283.09613 | [M±H]⁺/⁻ | ± | N/A | – | **+** | **+** | **+** | **+** | **+** |
| 23 | *5,7-Dimethoxy-6-C-methylflavone (form B)* | **Flavones** | C₁₈H₁₆O₄ | 5.52 | 295.09793 | [M−H]⁻ | − | N/A | – | – | **+** | – | – | – |
| 24 | *6-Methylflavone* | **Flavones** | C₁₆H₁₂O₂ | 7.61 | 237.09070 | [M+H]⁺ | + | 9.4 | – | **+** | – | **+** | – | – |
| 25 | *Dimethylcaffeic acid* | **Hydroxycinnamic Acids** | C₁₁H₁₂O₄ | 3.00 | 191.07028 | [M+H]⁺ | + | 85.8 | – | – | **++** | **++** | **++** | **+++** |
| 26 | *Ferulic acid* | **Hydroxycinnamic Acids** | C₁₀H₁₀O₄ | 4.29 | 193.05078 | [M−H]⁻ | − | N/A | – | – | – | **+** | **+** | **+** |
| 27 | *4-Methoxycinnamic acid* | **Hydroxycinnamic Acids** | C₁₀H₁₀O₃ | 4.78 | 177.05594 | [M−H]⁻ | − | N/A | – | – | – | – | **+** | **+** |
| 28 | *7-Methoxy-2-methylisoflavone (form A)* | **Isoflavones** | C₁₇H₁₄O₃ | 7.24 | 265.08728 | [M−H]⁻ | − | N/A | **++** | **++** | **++** | **++** | **++** | **+** |
| 29 | *7-Methoxy-2-methylisoflavone (form B)* | **Isoflavones** | C₁₇H₁₄O₃ | 6.50 | 289.08312 | [M+H]⁺ | + | N/A | **++** | **++** | **++** | **+** | **+** | **+** |
| 30 | *3'-Methoxydihydroformononetin* | **Isoflavones** | C₁₇H₁₆O₅ | 8.87 | 281.08222 | [M−H]⁻ | − | N/A | **+** | – | **+** | **+** | **+** | **+** |
| 31 | *4'-Hydroxy-7-methoxy-8-methylflavan* | **Other Polyphenols** | C₁₇H₁₈O₃ | 8.91 | 269.11860 | [M−H]⁻ | − | N/A | – | – | **+** | **++** | **++** | **++** |
| 32 | *4,4'-Dihydroxybenzophenone* | **Phenylpropanoids/Simple Phenols** | C₁₃H₁₀O₃ | 5.68 | 213.05599 | [M±H]⁺/⁻ | ± | 9.8 | **++** | **++** | – | **++** | **++** | **++** |
| 33 | *4-Hydroxybenzaldehyde* | **Phenylpropanoids/Simple Phenols** | C₇H₆O₂ | 3.29 | 121.02975 | [M±H]⁺/⁻ | ± | 10.0 | – | **+** | **++** | **++** | **++** | **++** |
| 34 | *1-(2,4-Dihydroxyphenyl)-2-(3,5-dimethoxyphenyl)propan-1-one* | **Phenylpropanoids/Simple Phenols** | C₁₇H₁₈O₅ | 5.80 | 301.10848 | [M−H]⁻ | − | 53.0 | **+** | **+** | – | **++** | **++** | **++** |
| 35 | *2,4-Bis(4-hydroxybenzyl)phenol* | **Phenylpropanoids/Simple Phenols** | C₂₀H₁₈O₃ | 8.85 | 305.11857 | [M−H]⁻ | − | 9.5 | **+** | **+** | – | **+** | – | – |
| 36 | *1,3-Bis(2,4-dihydroxyphenyl)propane* | **Phenylpropanoids/Simple Phenols** | C₁₅H₁₆O₄ | 5.01 | 259.09787 | [M±H]⁺/⁻ | ± | 26.5 | **+** | – | **+** | **+** | **++** | **++** |
| 37 | *4-Hydroxybenzophenone* | **Phenylpropanoids/Simple Phenols** | C₁₃H₁₀O₂ | 4.76 | 197.06100 | [M−H]⁻ | − | 9.5 | – | – | – | **+** | **++** | **++** |
| 38 | *Catechol* | **Phenylpropanoids/Simple Phenols** | C₆H₆O₂ | 2.23 | 109.02978 | [M−H]⁻ | − | 10.0 | – | – | **+** | **+** | **++** | **++** |
| 39 | *Diorcinol* | **Phenylpropanoids/Simple Phenols** | C₁₄H₁₄O₃ | 5.00 | 229.08726 | [M±H]⁺/⁻ | ± | 32.5 | – | – | **+** | **+** | **+** | **+** |
| 40 | *Diorcinol H* | **Phenylpropanoids/Simple Phenols** | C₁₅H₁₄O₄ | 7.44 | 257.08227 | [M−H]⁻ | − | N/A | – | – | **+** | **+** | **+** | **+** |
| 41 | *3-Methoxyphenylacetic acid* | **Phenylpropanoids/Simple Phenols** | C₉H₁₀O₃ | 4.93 | 165.05587 | [M−H]⁻ | − | N/A | – | – | **+** | **+** | **+** | **+** |
| 42 | *3-(4-Hydroxyphenyl)-1-propanol* | **Phenylpropanoids/Simple Phenols** | C₉H₁₂O₂ | 3.19 | 153.09088 | [M+H]⁺ | + | N/A | – | – | – | **+** | **+** | **+** |
| 43 | *7-(4-Hydroxyphenyl)-6,7-dihydrofuro[3,2-g]chromen-5-one* | **Phenylpropanoids/Simple Phenols** | C₁₇H₁₂O₄ | 6.51 | 279.06656 | [M−H]⁻ | − | 26.4 | **+** | **+** | – | **+** | **+** | **+** |
| 44 | *1,8-Dihydroxy-10-[3-(4-hydroxyphenyl)propanoyl]anthracen-9-one* | **Phenylpropanoids/Simple Phenols** | C₂₃H₁₈O₅ | 7.23 | 373.10790 | [M±H]⁺/⁻ | ± | N/A | – | – | **+** | – | **+** | **+** |
